# Population Genetics of the Emergence and Evolution of Allogenic Recognition During Fertilization

**DOI:** 10.3390/biom15101397

**Published:** 2025-09-30

**Authors:** Masahiro Naruse, Takako Saito, Midori Matsumoto

**Affiliations:** 1 Acquisition, Technology & Logistics Agency, Shinjuku-ku 162-8870, Japan; naruse.masahiro.qb@ext.atla.mod.go.jp; 2 Department of Applied Life Sciences, Faculty of Agriculture, Shizuoka University, Shizuoka 422-8529, Japan; saito.takako@shizuoka.ac.jp; 3 Research and Education Center for Natural Science, Keio University, Yokohama 223-8521, Japan

**Keywords:** allorecognition, evolution, fertilization, population genetics

## Abstract

Allorecognition, or distinguishing between the self and nonself within the same species, is observed in both animals and plants, particularly in the context of immune reactions and self-incompatibility in sexual reproduction. Polymorphic recognition molecules are known to be responsible for such allorecognition during fertilization. Previous studies have reported that in ascidians and flowering plants, inbreeding avoidance relies on a pair of polymorphic recognition molecules with a receptor-ligand relationship that are encoded at a single locus, the *S* locus (Self-incompatibility locus), but the process by which such pairs of recognition molecules emerge and evolve to become polymorphic is not known. Here, a population genetics study was carried out as a novel approach for investigating allorecognition. To study the process by which self-recognition emerges, we simulated a situation in which an allorecognizing genotype is generated from a nonallorecognizing genotype through mutation and then analyzed whether the two genotypes could coexist. The conditions under which the numbers of allorecognition alleles could increase over evolutionary time were investigated, and the generational dynamics of nonallorecognizing genotypes were analyzed. Subsequent modeling was carried out to reproduce the allorecognition mechanism in *Ciona*, and consistency between the simulation results and experimental data was observed. Our approach provides new insight into the evolutionary process of allorecognition.

## 1. Introduction

Fertilization, or gamete fusion, is a pivotal event in sexual reproduction in animals and plants. In general, a male gamete, or sperm, is released and attracted to a female gamete, or egg. Whereas most flowering plants are hermaphrodites, a majority of the animals are dioecious, with some exceptions, including ascidians. Among angiosperms, approximately half of all species, including rice and *Arabidopsis*, are self-compatible [1,2,3]. Similarly, both self-incompatible and self-compatible species of ascidians have been characterized: *Ciona intestinalis* and *Halocynthia roretzi* are well-known self-incompatible species, whereas *Phallussia mammillata* is self-compatible [4,5].

Self-compatibility appears to be advantageous for population growth, since it allows female gametes to be fertilized with male gametes from the same animal even in the absence of a mating partner: a good example of a self-compatible animal species is *Caenorhabditis elegans* [6]. On the other hand, individuals produced by self-fertilization have lower genetic diversity than those produced by nonself-fertilization, and as a result, self-fertilization causes inbreeding depression [7]. For example, in *Ciona*, self-fertilization results in the production of only a small number of normal embryos and larvae [8]. Self-fertilization in hermaphroditic colonial tunicates of the genus *Botryllus* also results in the production of an extremely small number of progeny due to embryonic death [9]. Therefore, although the prevention of self-fertilization has a certain disadvantage in terms of reducing opportunities for reproduction, it appears to have a significant advantage in eliminating harmful genes and promoting the spread of beneficial genes.

Although some species avoid self-fertilization by modulating reproductive behavior, such as by releasing male and female gametes at different times [10,11], many species prevent self-fertilization through allogenic recognition (allorecognition) mechanisms. In *Ciona intestinalis* type A (another name *Ciona robusta*), sperm can recognize eggs from the same individual upon attachment to the vitelline coat (VC), causing detachment from the VC or eventual cessation of motility, ultimately preventing self-gamete fusion. The sperm-VC recognition protein s-Themis, a PKD-like transmembrane protein, and the VC protein v-Themis, a fibrinogen-like protein, play key roles in this process [4]. Three pairs of *s-Themis* and *v-Themis* genes, *s*/*v-Themis-A*, *s*/*v-Themis-B*, and *s*/*v-Themis-B2*, are found at two loci: *s*/*v-Themis-A* on chromosome 2q (locus A) and *s*/*v-Themis-B* and *s*/*v-Themis-B2* on chromosome 7q (locus B). In every case, *v-Themis* genes are located in the first intron of *s-Themis* genes but are transcribed in the opposite direction [4,5,12,13]. If three allelic pairs (A, B and B2) of s-Themis and v-Themis are matched, i.e., the same haplotype, fertilization is prevented, enabling self-incompatibility [12].

Similarly, in Papaveraceae, if self-pollen reaches the stigma, pollen germination and pollen tube elongation do not occur, and as a result, self-fertilization never takes place. The pollen recognition protein PrpS and the stigmatic recognition protein PrsS, genes of which are encoded in a single diverse genetic locus, the *S*-locus, in close proximity (within 0.5 kbp) to one another, are responsible for self-incompatibility in Papaveraceae [1,14].

In both cases, male and female recognition molecules, encoded by genes at one or a few loci, play pivotal roles in ligand-receptor interactions. When VC or stigmatic ligand molecules interact with receptors on self-sperm or self-pollen, respectively, intracellular Ca^2+^-mediated signal transduction occurs, resulting in the prevention of self-fertilization [15,16]. Both male and female allorecognition proteins are thought to be highly polymorphic and mediate specific interactions [17]. The polymorphic alleles responsible for such allorecognition are presumed to have originated from a single ancestral gene, encoding a pair of ancestral male and female recognition molecules believed to have diverged to produce polymorphisms during coevolution [18].

Here, we investigated the emergence and evolution of allorecognition molecules using a population genetics approach. For the emergence process, we evaluated whether a primitive self-recognition gene that emerged by mutation was maintained from generation to generation. The process, by which a pair of self-recognition proteins acquire variation by mutation was then simulated using a model in which a male or female mutation preceded the creation of a new allorecognition gene pair. Finally, we examined how the proportions of allorecognition alleles and nonallorecognition alleles changed when the number of allorecognition alleles or loci increased.

## 2. Materials and Methods

For this study we considered three preconditions for our modeling, and the model represents phenomena that occur in animals. Therefore, the sperm and the egg in the animal model correspond to the pollen and the pistil in plants, respectively. 

The first precondition is that the pair of allorecognition molecules is specialized for fertilization and is not shared with other types of allorecognition, such as nonself recognition in the immune system. At least in the ascidian, *Halocynthia roretzi*, different molecular mechanisms have been suggested for fertilization and the immune response, since the characteristics of self-incompatibility and allogenic refusion of somatic cells (contact reaction) differ [19,20]. Additionally, the Fu/HC gene, which plays a key role in histocompatibility, was not expressed in germline cells in the ascidian *Botryllus schlosseri* [21].

The second precondition is that male recognition molecules are expressed from haploid cells, whereas female recognition molecules are derived from diploid cells, whether from eggs arrested at the first metaphase of meiosis in ascidians or from the somatic cells surrounding eggs, as reported in *C. intestinalis* [5,22]. In plants, this type of self-recognition in mating is called gametophytic, distinguished from sporophytic, in which male recognition molecules are synthesized in the anther tapetum (somatic cells) and attached to the surface of the pollen, although both gametophytic and sporophytic self-recognition have been reported [1,23].

The third precondition for modeling was that neither mutations nor gene migration occured during the simulation period and that generations did not overlap, as is typical in the modeling of population genetics [24]. This is to focus our examination on each step of emergence and the evolution of allorecognition, which consists of the appearance of a certain new allele by mutation and subsequent convergence to equilibrium through the spread of the selection of alleles in the population, rather than simulating the mutation process itself in real-time. In the simulation, genotypes compete through natural selection rather than genetic drift.

### 2.1. Modeling for the Emergence Process of Allorecognition Genes

**Figure 1:**
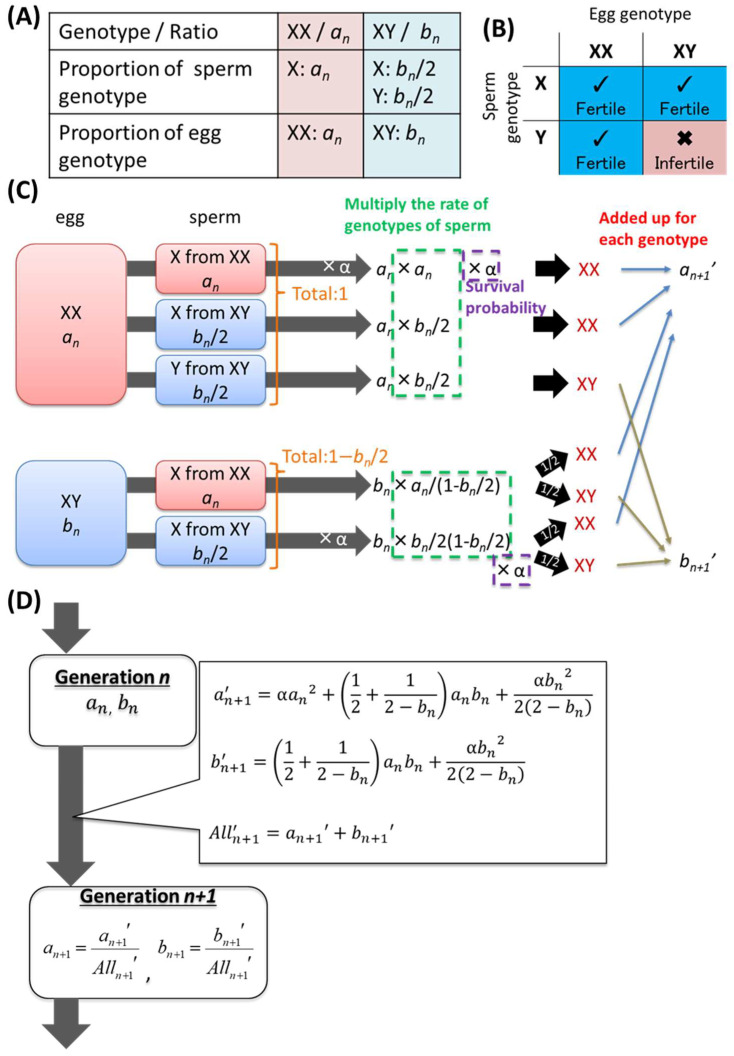
Model for the process of self-recognition gene emergence. (**A**) Definitions of the genotypes and alleles in the model. (**B**) Fertilization characteristics of genotypes in the model. X-genotype sperm can fertilize any egg (✔ Fertile). Y-genotype sperm can not fertilize XY-genotype eggs (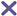 Infertile). (**C**) Calculation of the next generation in the model. (**D**) Flow and equations for the calculation of the *n*+1-st generation from the *n*-th generation.

The model for the process of self-recognition gene emergence is shown in Figure 1. X represents a nonallorecognition allele, Y represents an allorecognition allele that emerged as a result of the mutation, and $a_{n}$ and $b_{n}$ indicate the proportions of individuals with the XX and XY genotypes, respectively, in the *n*-th generation, where $a_{n}+b_{n}=1$, because the YY genotype does not exist (as described below).

Since the numbers of sperm and eggs should be proportional to the number of individuals, $a_{n}$ X-genotype sperm are produced from XX individuals, while $b_{n}/2$ X-genotype and $b_{n}/2$ Y-genotype sperm are produced from XY individuals (Figure 1A). Similarly, the ratio of XX-genotype eggs to XY-genotype eggs can be regarded as $a_{n}$ vs. $b_{n}$. Although X-genotype sperm can fertilize any egg, sperm carrying the Y allele can fertilize only XX-genotype eggs (Figure 1B).

Assuming that all the eggs undergo fertilization with an excess amount of sperm, the number of offspring in the next generation produced by each combination of gametes can be expressed as the product of the proportions of eggs and sperm, as shown in Figure 1C. Here, YY-type offspring cannot occur, since Y-type sperm cannot fuse with XY-type eggs. Before the number of offspring for each genotype is summed, the survival probability α must be adjusted if the parental genotypes are the same because inbreeding depression must be considered for offspring of pairs of the same genotype, i.e., self-fertilized offspring. For cases of inbreeding depression, the value of α should be between 0 and 1, where α=0 indicates that inbreeding is completely lethal, α=1 indicates that inbred and outcrossed offspring exhibit equal survival, and α>1 indicates that inbred offspring have greater survival than outcrossed offspring do, which is unlikely. Finally, the sums of each genotype are defined as ${a_{n+1}}^{\prime }$ and ${b_{n+1}}^{\prime }$, respectively; then, $a_{n+1}$ and $b_{n+1}$, which are normalized values obtained by dividing each value by their sum, defined as ${{All}_{n+1}}^{\prime }$, are the proportions of XX and XY genotype individuals of the *n*+1st generation (Figure 1D).

For the simulation, $a_{0}$ and $b_{0}$ were set to arbitrary initial values, and the generation calculation in Figure 1D was simply repeated as many times as necessary for the analysis. The reproducibility and robustness of the convergence points of each population were confirmed by the simulation using randomized initial conditions; the same was applied to the following models.

### 2.2. Modeling of the Evolution of Allorecognition Genes

#### 2.2.1. Divergence Through Mutation

**Figure 2:**
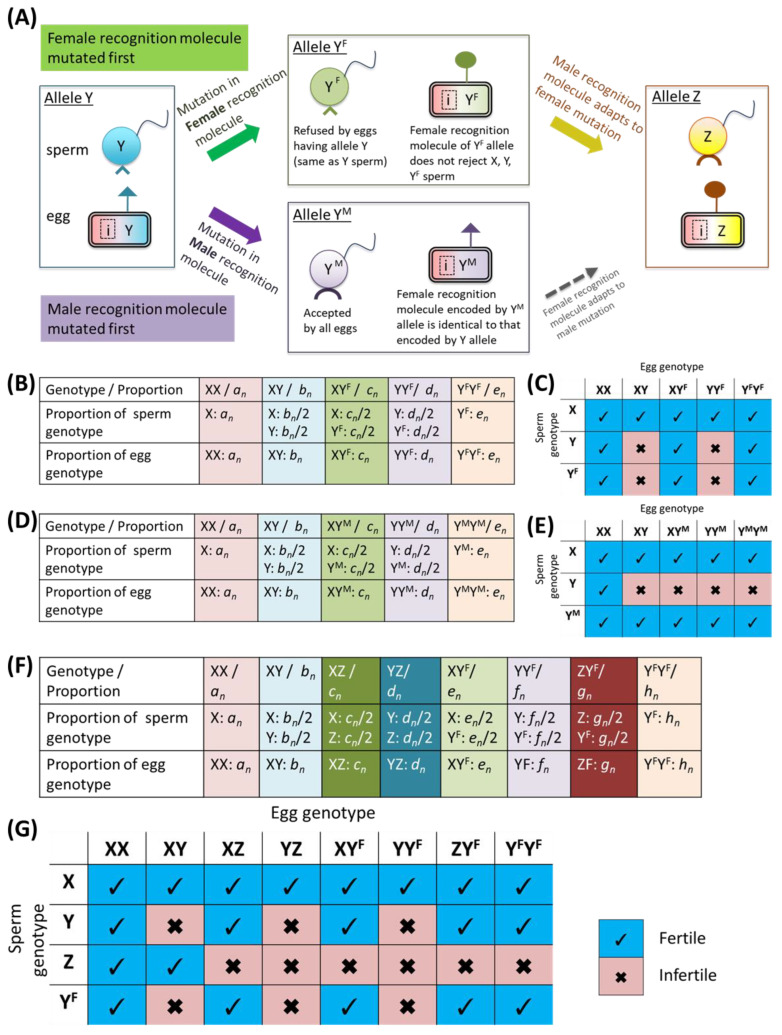
Models for the divergence of the allorecognition locus. (**A**) Two possible routes for the creation of the new allele and allele definitions. In the upper route, mutation in the gene encoding the female recognition molecule occurs first, after which the gene encoding the male recognition molecule adapts to this change through its own mutation. In contrast, in the lower route, the male gene is mutated first. “i” in the egg represents any other allele; inhibition of fertilization by such an allele is not described here. (**B**) Definitions of the genotypes and alleles in the model, comprising alleles X, Y, and Y^F^. (**C**) Characteristics of genotypes in the model, comprising alleles X, Y, and Y^F^. (**D**) Definitions of the genotypes and alleles in the model, comprising alleles X, Y, and Y^M^. (**E**) Characteristics of genotypes in the model, comprising alleles X, Y, and Y^M^. (**F**) Definitions of the genotypes and alleles in the model, comprising alleles X, Y, Z, and Y^F^. (**G**) Characteristics of genotypes in the model, comprising alleles X, Y, Z, and Y^F^.

To study the evolutionary process of allorecognition genes, namely, the generation of variation in allorecognition alleles, the process of allorecognition allele divergence must be considered first. Thus, situations in which a mutation occurred in a gene encoding the male or female recognition molecule of a primitive allorecognition gene in the previous model (Figure 1) were modeled as shown in Figure 2, Appendix A.1, and Supplementary Material S1.

In addition to the definition in Figure 1, we defined an allele Y^F^, in which a mutation occurred in a gene encoding the female recognition molecule on allele Y and whose function was lost, and an allele Y^M^, in which a mutation occurred in a gene encoding the male recognition molecule (Figure 2A). A mutated female recognition molecule derived from the Y^F^ allele does not lead to fertilization failure for sperm with a male recognition molecule encoded by either the Y allele or the Y^F^ allele, whereas the sperm of Y^F^ cannot fertilize eggs with the Y allele. Therefore, this mutation results in one-way sterility. With respect to the Y^M^ allele, sperm with a mutated male recognition molecule derived from the Y^M^ allele are permitted to fertilize eggs with the Y allele or Y^M^ allele, whereas eggs with the Y^M^ allele cannot be fertilized by sperm with the Y allele, similar to eggs with the Y allele. Models for alleles Y^F^ or Y^M^ were subsequently constructed (Figure 2B,D, respectively), and the mating characteristics of the genotypes in these models are shown in Figure 2C,E.

On the basis of these features, the relationships of the proportions of the *n*-th generation and the *n*+1-st generation were derived as Equations (A1) and (A2) through works similar to those in Figure 1C,D (see Figure S1A,B). Changes in the population occupancy rates of the Y^F^ allele or the Y^M^ allele were analyzed by repeating these generation calculations.

For further analysis of the divergence process, a situation in which a new allele encoding a male recognition molecule adapted to the mutated female molecule appears, must be considered. This new allele was defined as allele Z, and the populations consisting of X, Y, Z and Y^F^ were modeled as shown in Figure 2F. In this case, the XX, XY, XZ, YZ, XY^F^, YY^F^, ZY^F^, and Y^F^Y^F^ genotypes are present.

Only the X-type sperm can be accepted by any egg, the Y-type sperm can be fertilized with only the XX- and XZ-type eggs, and the Z-type and Y^F^-type sperm can fertilize XX, XY, XY^F^, YY^F^, and Y^F^Y^F^ genotype eggs (Figure 2G). On the basis of these features, the relationship between the proportions of the *n*-th generation and the *n*+1-st generation was derived by Equation (A3) through a process similar to those in Figure 1C,D (see Figure S1C). Changes in the population occupancy rates of alleles X, Y, Y^F^, and Z were simulated by repeating the generation calculations.

#### 2.2.2. Model with Divergent Alleles

**Figure 3:**
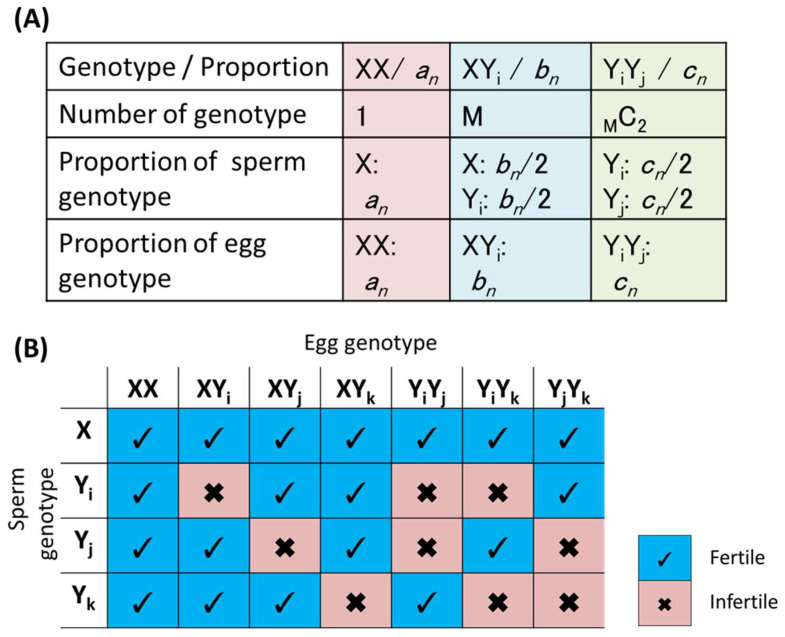
Model with “M” types of allorecognition alleles. (**A**) Definitions of the genotypes and alleles in the model. (**B**) Characteristics of the genotypes in the model.

To simulate a situation in which the number of allorecognition alleles increases, it is necessary to consider three groups of genotypes, namely, a homozygous genotype of the nonallorecognition allele, a heterozygous genotype comprising the no allorecognition allele and one of the allorecognition alleles, and a heterozygous genotype comprising two different allorecognition alleles (a, b, and c in Figure 3A, respectively).

As shown in Supplementary Material S2, a preliminary study in which two types of allorecognition alleles (Y_1_ and Y_2_ in Figure S2A) coexisted with the nonallorecognition allele X (defined as M=2) revealed that heterogenotypic populations of a nonallorecognition allele and one of the allorecognition alleles (${b^{1}}_{n}$ and ${b^{2}}_{n}$ in Figure S2C,D), placed under the same conditions, converged to the same proportion as the number of generations increased, even if the simulations started from different initial proportions (${b^{1}}_{0}$ and ${b^{2}}_{0}$). Additionally, a preliminary study with three types of allorecognition alleles (defined as M=3) revealed that the heterogenotypic populations consisted of two different allorecognition alleles (${c^{1}}_{n}$, ${c^{2}}_{n}$, and ${c^{3}}_{n}$ in Figure S2G,H) converged to the same proportion, since these genotype populations were selected under the same conditions.

On the basis of the results of these preliminary studies, the simulation model was extended to M>3 and generalized. The characteristics of the model with “M” types of allorecognition alleles are shown in Figure 3. Here, heterogenotypic populations under the same conditions were substituted as ${b^{1}}_{n}={b^{2}}_{n}={b^{3}}_{n}=\ldots ={b^{M}}_{n}=b_{n}$ and ${c^{1}}_{n}={c^{2}}_{n}={c^{3}}_{n}=\ldots ={c^{{\mathrm{MC}}_{2}}}_{n}=c_{n}$ (Figure 3A). Afterward, the relationship between the proportions of the *n*-th generation and the *n*+1-st generation was derived by Equation (A4) through procedures similar to those in Figure 1C,D (see Supplementary Material S3). Changes in the occupancy rates of alleles X, and Ys were simulated by repeating the generation calculations.

### 2.3. Modeling the 2-Locus Situation

**Figure 4:**
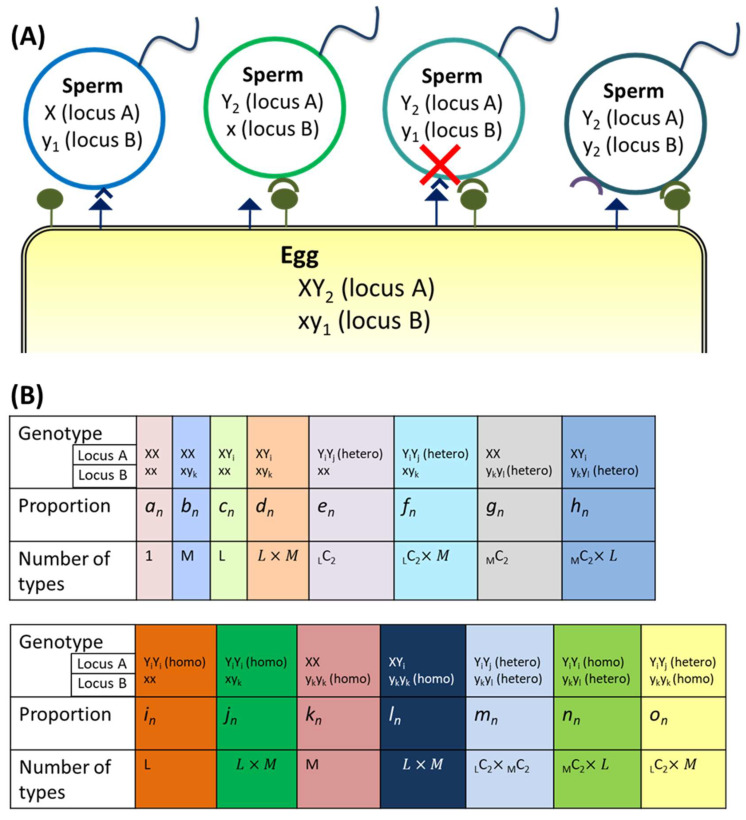
Model with two loci. (**A**) Recognition between gametes in the 2-locus model. In this model, the egg rejects only the sperm that has the same alleles as the egg at both loci (red cross), although sperm that share only one allele at one locus with the egg can fertilize the egg. (**B**) Definitions of the genotypes and alleles in the model.

To investigate the effects of the presence of multiple allorecognition loci, a 2-locus model was constructed according to the characteristics of *C. intestinalis*. To simulate the fertilization of *C. intestinalis*, sperm cannot fertilize eggs that share the same alleles at all the loci (Figure 4A).

The definitions of the genotypes and their characteristics are described in Figure 4B and Figure S4A. Loci A and B have “M” and “L” types of allorecognition alleles, respectively. As in the model with divergent alleles above, genotypes under the same conditions were combined; e.g., $b_{n}$ represents M types of populations, with homononallorecognition in locus A and heteroallorecognition in locus B, under the same conditions.

The relationships between the proportions of the *n*-th generation and the *n*+1-st generation were subsequently derived by Equation (A5) through a process similar to that in Figure 1C,D. Changes in the population occupancy rates were simulated by repeating the generation calculation.

## 3. Results

### 3.1. Emergence Process of Allorecognition Genes

Using a model for the emergence process described in the Materials and Methods (Figure 1), we observed the proportions of genotypes across generations.

**Figure 5:**
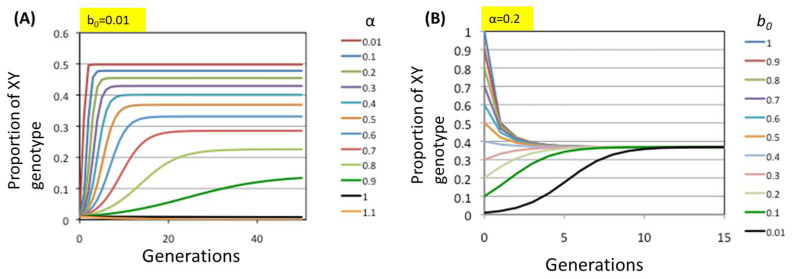
Representative results of simulations of the process of allorecognition gene emergence. The proportions of XY individuals (vertical axis) were plotted against the number of generations (horizontal axis). (**A**) Results of the cases where the initial proportion of XY individuals was fixed at $b_{0}=0.01\;$ and α was varied between 0.01 and 1.1. (**B**) Results of the cases where α was fixed at 0.2 and the initial proportion of XY individuals was varied between 0.01 and 1. In all the cases, the proportions of XY individuals converged to the same value, approximately 0.37, after 5–10 generations.

The changes in the proportion of XY genotype individuals ($b_{n}$) across generations when the initial value $b_{0}$ is fixed at 0.01 and the survival probability α varies between 0.01 and 1.1 are shown in Figure 5A. As shown in Figure 5A, $b_{n}$ converges to a certain value when 1>α>0, whereas it converges to 0 for α>1 and decreases monotonically for α=1, indicating that genotypes XX and XY coexist as long as 1>α.

As shown in Figure 5B, when α is fixed at 0.2, simulations with any initial value $b_{0}$ yielded convergence of the XY proportion at the same value of approximately 0.45 after multiple generations. In other cases where α<1, the proportion of XY genotype individuals which converged over generations was dependent on only α regardless of the $b_{0}$ values, and reproducibility was confirmed by the simulation using randomized initial conditions, as shown in Supplementary Material S5.

In conclusion, the allorecognition allele can be fixed in the population at a certain proportion that depends on α. In such a situation, the allorecognition allele (Y) and nonallorecognition allele (X) are in a state of balanced selection. Although heterozygous dominance is known to lead to balanced selection as in the case of sickle-cell polymorphisms in malaria-endemic regions [25,26], the relationship between X and Y here involves a different mechanism.

### 3.2. Process of Allorecognition Gene Evolution

#### 3.2.1. Process of Divergence Through Mutations in Male/Female Genes

When polymorphism of allorecognition alleles occurs, it is unlikely that a gene pair encoding male and female recognition molecules will simultaneously undergo mutation to generate a new gene pair that encodes a pair of functional recognition molecules. Therefore, it is natural to hypothesize that mutation occurs first within only one sex (gene encoding a male or female recognition molecule), followed by adaptive mutation of the gene encoding the corresponding molecule. Under this hypothesis, we simulated situations in which only the male or female allorecognition molecule underwent loss-of-function mutation via the models described in the Materials and Methods (Figure 2), and the resulting changes in the allele occupancy rates were determined.

**Figure 6:**
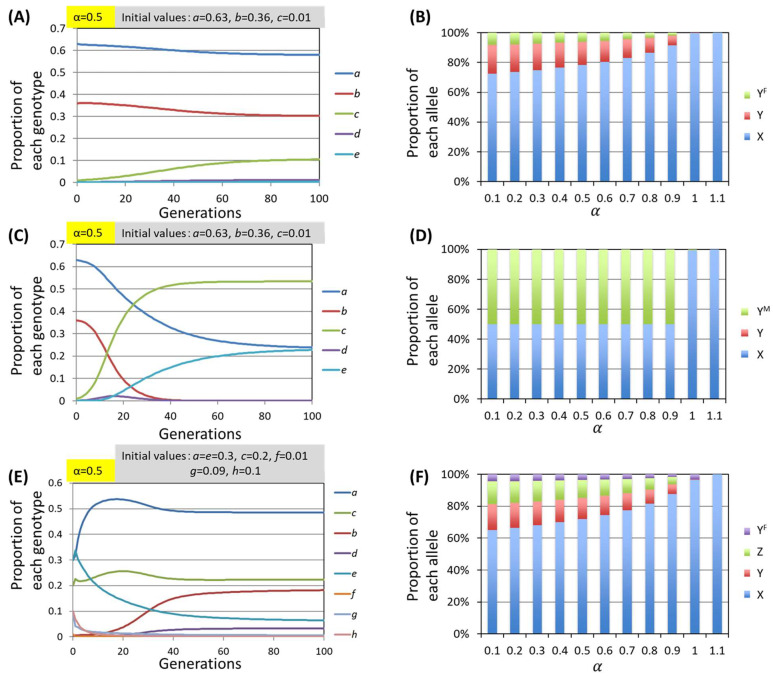
Results of the simulation of the allorecognition locus divergence process. (**A**,**C**,**E**) Representative simulation results. The proportions of each genotype (vertical axis) were plotted against the number of generations (horizontal axis). The initial proportions of each genotype are listed in each figure. α was fixed at 0.5. (**B**,**D**,**F**) Summarized results of the simulations. The proportions of each allele in the populations (vertical axis) were plotted against the value of α (horizontal axis). (**A**,**B**) Results of the model with alleles X, Y, and Y^F^. (**C,D**) Results of the model with alleles X, Y, and Y^M^. After multiple generations, only the Y allele was eliminated from the population when 1>α. (**E**,**F**) Results of the model with alleles X, Y, Z and Y^F^.

First, when the Y^F^ allele emerges from the mutation in the population consisting of alleles X and Y, the Y^F^ allele is fixed in the population at a certain rate, and the population transitions to a state comprising X, Y, and Y^F^ alleles when α<1. At this time, the converged proportion values of each genotype depended not on the initial value but rather on α. Representative results of the simulations are shown in Figure 6A, and the summarized results of the simulation at convergence are shown in Figure 6B. These results indicate that the Y^F^ allele coexists with the X and Y alleles.

In contrast, when the case in which the Y^M^ allele appears in the population consisting of alleles X and Y is simulated, the Y allele tends to disappear from the population. As shown in a representative result (Figure 6C), genotypes with the Y allele, namely, XY and YY^M^, converged to 0 in the presence of the Y^M^ allele at α=0.5. The summarized results of the simulations (Figure 6D) show the converged proportion values of each genotype for various α values, indicating that the Y allele does not survive in all the cases. Therefore, the elimination of the original Y allele occurs through mutation of the gene encoding the male recognition molecule on Y allele.

Considering the evolution of polymorphisms in the allorecognition gene, we focus first on the case in which the mutation occurs in the gene encoding the female recognition molecule, since this case can be a transition state to the creation of a new allorecognition allele, whereas mutation in the male gene leads to the disappearance of the original allorecognition allele.

To create a new allorecognition allele, the male recognition molecule must adapt to the mutated female recognition molecule encoded by the Y^F^ allele. This requires a mutation in the gene encoding the male recognition molecule of allele Y^F^, and an allele that accomplishes such a mutation is defined as allele Z, as shown in Figure 2A. Therefore, simulations for the state comprising alleles X, Y, Z and Y^F^ were conducted using the model described in the Materials and Methods (Figure 2).

An example of the results is shown in Figure 6E; the proportions of each genotype tended to converge to a fixed value, and none of them were eliminated when α<1. The convergence proportions depend on α regardless of the initial value, as in previous simulations. The relationships between α and the proportions of alleles after different numbers of generations are shown in Figure 6F, indicating that all the genotypes coexist in all the cases where α≥1. A comparison of the data in Figure 6B,F revealed that the proportion of Y^F^ allele was significantly reduced by the emergence of the Z allele even at the same α.

The simulations in this section suggest that the creation of the new allorecognition allele consists of two steps. First, one of the existing alleles is mutated, and a “transition allele” encoding a new female recognition molecule and the original male recognition molecule is generated and can be maintained in the population. In the second step, a “new allorecognition allele” encoding a male recognition molecule that can recognize the new female recognition molecule, encoded by the transition allele, is generated by the mutation and coexists in the population, accompanied by a significant reduction in the proportion of transition alleles, which ultimately can be eliminated by genetic drift.

#### 3.2.2. Behavior of the Model with Divergent Alleles

In the above studies, allorecognition alleles and nonallorecognition allele coexisted across generations, which was related to balancing selection. Next, we focused on how this relationship changes when the number of allorecognition alleles increases.

Using the models described in the Section 2 (Figure 3), the proportion of nonallorecognition allele after different numbers of generations was simulated in the presence of multiple allorecognition alleles. When the number of types of allorecognition alleles is defined as “M”, there are M types of heterozygous genotypes of the nonallorecognition allele and one of the allorecognition alleles (“$b_{n}$”) and _M_C_2_ types of heterozygous genotypes of the two different allorecognition alleles (“$c_{n}$”), as described in Figure 3A.

**Figure 7:**
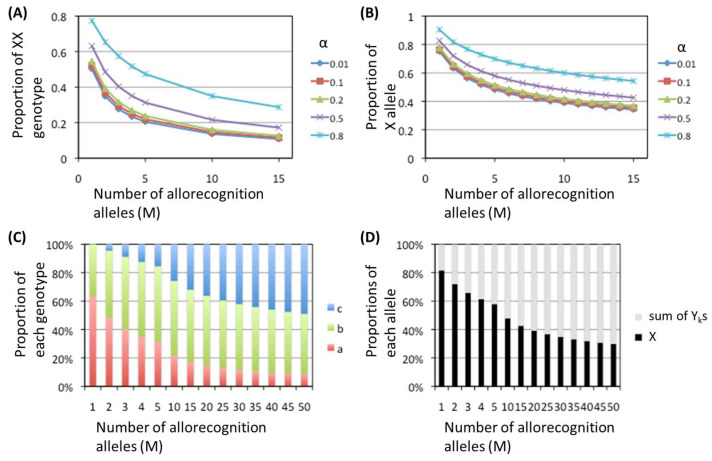
Summarized results of the simulation using the model with divergent alleles. (**A**) The proportions of XX individuals that converged across generations (vertical axis) plotted against the number of allorecognition alleles (M), up to M = 15; α varied between 0.01 and 0.8. (**B**) The proportions of X alleles that converged across generations (vertical axis) plotted against the number of allorecognition alleles (M) up to M = 15; α was varied between 0.01 and 0.8. (**C**) Proportions of each genotype that converged across generations (vertical axis) plotted against the number of allorecognition alleles (M) up to M = 50; α was fixed at 0.5. (**D**) Proportions of nonallorecognition allele X and the sum of allorecognition alleles (Y_k_s) converging across generations (vertical axis) plotted against the number of allorecognition alleles (M) up to M = 50; α was fixed at 0.5.

The results of the simulation up to M = 50 are summarized in Figure 7. The proportions of the XX genotype at the point of convergence against M for several α values are shown in Figure 7A, indicating that even when M increases to 15, the XX genotype still accounts for a certain percentage of the whole population. Even if α=0.01, meaning that cross-fertilization between the different genotypes is 100 times more advantageous for survival than fertilization between the same genotypes, more than 10% of the populations exhibited the XX genotype at M = 15. Additionally, a large proportion of individuals possessed the nonallorecognition allele X alone (Figure 7B). Further simulation up to M = 50 (Figure 7C,D) revealed that the proportion of the XX genotype and the X allele occupancy rate decreased monotonically with increasing M, although the decrease slowed. The data in Figure 7C,D indicate that even when M increases, a considerable number of nonallorecognition allele remains in heterozygosity with allorecognition alleles (“*b*” in Figure 7C).

Therefore, as the number of allorecognition alleles increases, the occupancy rate of the nonallorecognition allele decreases, but the allele survives in the population, mainly in heterozygosity with allorecognition alleles, and direct selection does not occur. These findings suggest that there is a high likelihood that nonallorecognition allele persists in populations of animals and plants that avoid self-mating by allorecognition.

### 3.3. Simulation Using the 2-Locus Model

In *C. intestinalis*, three gene pairs encoding allorecognition molecules, *s*/*v-Themis-A* in locus A and *s*/*v-Themis-B* and *s*/*v-Themis-B2* in locus B, are similar to each other and have been suggested to share a common ancestral locus. In this section, we analyzed the effects of the presence of multiple allorecognition loci, especially the occupancy rates of nonallorecognition alleles at different loci.

**Figure 8:**
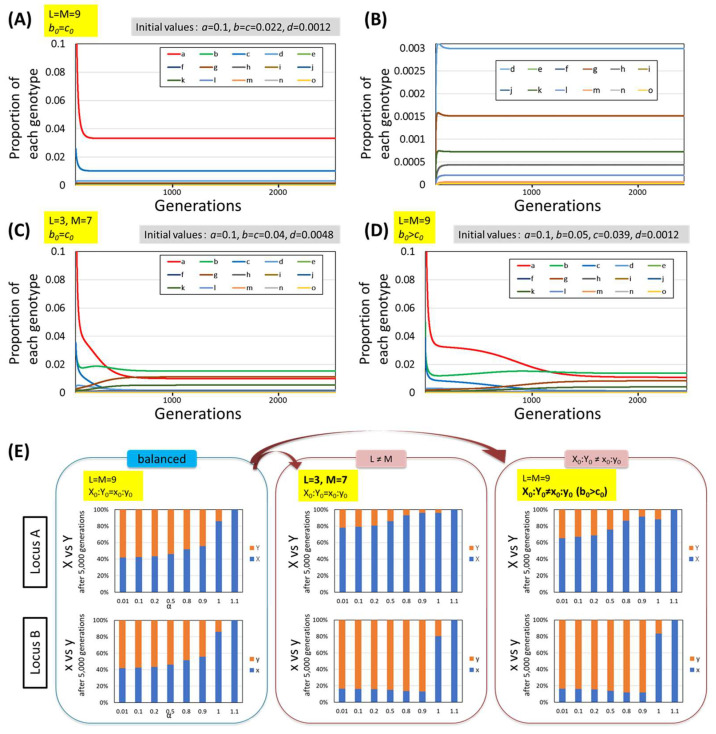
Results of the simulation using the 2-locus model. (**A**–**D**) Representative results of the simulation. The proportions of each genotype (vertical axis) were plotted against the number of generations (horizontal axis). L, M and the initial proportions are shown in each figure. α was fixed at 0.2. (**B**) Magnified chart of (**A**) showing the separation lower on the vertical axis. (**E**) Summarized result of the simulation. The ratios of X vs. Y at locus A and x vs. y at locus B after 5000 generations (vertical axis) were plotted against the value of α (horizontal axis). Here, Y and y represent the total proportions of allorecognition alleles at loci A and B, respectively. As shown in the left column (“balanced”), when both L=M and $X_{0}:Y_{0}=x_{0}:y_{0}$, X and Y at locus A and x and y at locus B coexist after many generations at α<1. When L=M was unbalanced, the proportion of nonallorecognition alleles at one locus (x) greatly decreased and that at the other locus (X) increased, after many generations (center column “L≠M”). A similar tendency was observed when $X_{0}:Y_{0}=x_{0}:y_{0}$ was unbalanced (right column “$X_{0}:Y_{0}\neq x_{0}:y_{0}$”).

A 2-locus model simulating *C. intestinalis* was constructed as described in the Materials and Methods (Figure 4), and changes in the occupancy rates of nonallorecognizing alleles were observed. The results of the simulations are shown in Figure 8, in which “L” is the number of alleles at locus A and “M” is the number of alleles at locus B. When the numbers of alleles at both loci were the same (L=M) and the initial proportions of the nonallorecognition alleles at both loci were equal ($X_{0}:Y_{0}=x_{0}:y_{0}$), all the genotypes, as defined in Figure 4B, coexisted after multiple generations (Figure 8A,B). However, when either L=M or $X_{0}:Y_{0}=x_{0}:y_{0}$ was unbalanced, the proportion of the nonallorecognition allele (X or x) at the locus with the greater number of allorecognition allele types (L and M) or the smaller initial proportion of the nonallorecognition allele was greatly reduced, trending toward disappearance. Figure 8C shows an example where L≠M (M > L), and Figure 8D shows a case where $X_{0}:Y_{0}\neq x_{0}:y_{0}$ ($b_{0}>c_{0}$). The proportion of nonallorecognition alleles, which decreased in this way, continued to decrease monotonically even after five thousand generations, suggesting a very high probability for the elimination of such nonallorecognition alleles through genetic drift. Such alleles are no longer in a state of balanced selection but rather in a state of direct selection. These reductions did not depend on the value of α (Figure 8E). In addition, in simulations of situations in which the nonallorecognition allele of one locus had disappeared, the nonallorecognition allele of the remaining other locus tended to persist (Supplementary Material S4 (Figure S4D–G)).

Therefore, when each of the two loci is polymorphic, the nonallorecognition allele tends to be eliminated at one locus, and survive at the other locus. In *C. intestinalis*, a nonallorecognition allele has been observed only at locus B (B2-3 allele in Ref. [12]), indicating that our model is consistent with observed phenomena.

## 4. Discussion

### 4.1. Validation of Simulations with Analytical Results

The reproducibility and robustness of the convergence points of each population were confirmed by the simulation using randomized initialization as shown in Table S1 and Supplementary Material S6. All the results showed consistent convergence to identical equilibrium states under various random initial conditions, indicating the reliability and robustness of the modeling.

The analytical solution of the model for the emergence process shown in Figure 1 was examined. When the proportion of the XX genotype converges, $a_{n}$ and $a_{n+1}$ should be equal. Therefore, the equilibrium points were calculated by substituting the equations in Figure 1C into the simultaneous equations $a_{n}=a_{n+1}$ and $a_{n}+b_{n}=1$. Then, real numbers between 0 and 1 satisfying Equation (1) are the equilibrium points for the model.

(1) $$\left(a_{n}-1\right)\left\{2\left(1-\alpha \right){a_{n}}^{3}+\left(5-4\alpha \right){a_{n}}^{2}+3\left(\alpha -1\right)a_{n}-\alpha \right\}=0$$

When 1 > α > 0, Equation (1) has two equilibrium points. Although one (a = 1) is an unstable equilibrium point, the other is a locally asymptotically stable equilibrium point such that the proportion of the genotype XX will converge to this stable equilibrium point regardless of the initial conditions. On the other hand, when α>1, a = 1 is the only equilibrium point of Equation (1), a state in which only the XX genotype exists. As shown in Table 1, the convergence points from the simulations match the stable equilibrium points obtained as the analytical solution.

Additionally, the analytical solution for the model with diversified alleles in Figure 3 was considered. As in the model for the emergence process, the following simultaneous Equation (2) were obtained by substituting the equations in Equation (A4) into simultaneous equations $a_{n}=a_{n+1}$, $b_{n}=b_{n+1}$, and an+Mbn+C2cn=1M . Then, “a” and “b”, which are between 0 and 1 and satisfy the equations in Equation (2), are the equilibrium points for the model.

(2) $$4\left(1-\alpha \right)a^{4}+2\left(1-\alpha \right)Ma^{3}b+4\left\{M\left(1-\alpha \right)+2\alpha -1\right\}a^{3}+2\left(1+\alpha \right)Ma^{2}b+\left(3-2\alpha \right)M^{2}ab^{2}\;+4\left\{\left(M-1\right)\alpha -1\right\}a^{2}+4M\left(M-1\right)ab+\left(M+\alpha -1\right)M^{2}b^{2}+4\left(M-1\right)a=0\;\begin{array}{cc} 8\left(1-\alpha \right)Ma^{4}b+ & 8\left(1-\alpha \right)M^{2}a^{3}b^{2}+2\left(1-\alpha \right)M^{3}a^{2}b^{3}-8a^{4}+4\left\{\left(3M-4\right)\left(1-\alpha \right)-3\right\}Ma^{3}b\\ & +2\left\{\left(3M-4\right)\left(1-\alpha \right)-3\right\}M^{2}a^{2}b^{2}+\left(3-4\alpha \right)M^{3}ab^{3}+2\left(1-\alpha \right)M^{4}b^{4}-8\left(2M-3\right)a^{3}\\ & -4\left(M-1\right)\left\{\left(M-7\right)+\alpha \left(M-2\right)\right\}Ma^{2}b-2\left(4M-\alpha \right)M^{2}ab^{2}+\left\{M-6-\alpha \left(2M-5\right)\right\}M^{3}b^{3}\\ & -8\left(M-1\right)\left(M-3\right)a^{2}-4M\left({2M}^{2}-4M+1\right)ab-\left\{2M^{2}+M-6-\left(M-2\right)\alpha \right\}M^{2}b^{2}\\ & +8{\left(M-1\right)}^{2}a+4M\left(M-1\right)b=0 \end{array}$$

As shown in Table 2, the convergence points from the simulations coincided with the stable equilibrium points obtained as the analytical solution. These alignments indicate that the simulation model and the approach of this study are appropriate for understanding the emergence and evolutionary processes in the allorecognition system.

An increase in the number of types of alleles leads to an increase in the probability of elimination of nonallorecognition alleles, especially if there are two or more loci. With respect to the one-locus situation, theoretically, when the number of allorecognition allele types (M) increases to infinity, the following simultaneous equations (Equation (3)) are obtained by considering only the highest-order terms, namely, the cubic and quartic terms of Equation (2).

(3) $$b^{2}=0\;\;2\left(1-\alpha \right)b^{4}+\left(1-2\alpha \right)b^{3}-2b^{2}=0$$

The solution of Equation (3) is b = 0. Since a = 1 or a = 0 satisfies Equation (2), (a, b, c) = (1, 0, 0) and (a, b, c) = (0, 0, 1/_M_C_2_) are equilibrium points. Whereas (1, 0, 0) is unstable, (0, 0, 1/_M_C_2_) is a stable equilibrium point. Therefore, as “M” continues to increase to infinity, the proportions of genotypes “a” and “b” converge to zero; that is, the nonallorecognition allele X will theoretically be eliminated from the population.

### 4.2. Comparison with Observed Phenomena

The simulations in this study suggest that the nonallorecognition allele may not be eliminated from the population; in nature, such an allele (B2-3 allele in *C. intestinalis*) exists and cannot perform the allorecognition function [12]. The sequence of this B2-3 allele is not particularly close to that of any of the other alleles of Themis-B2 (Supplementary Material S7), suggesting that the B2-3 allele has been independent for many generations.

The simulation indicates that the fitness of the nonallorecognition allele generally depends on α. There are no data comparing the fitness of offspring of the same allorecognition genotype with different genotypes, and the size of α may change depending on the size of the population, since the probability that mating between the same genotypes represents self-mating decreases with increasing population size. However, as mentioned above, in ascidians, self-fertilized embryos very often fail to develop [6,9], and abnormalities in seed formation caused by self-pollination have been reported in plants [27]. These observations indicate that self-fertilization carries considerable risk, and α must be very small.

### 4.3. Asymmetric Characteristics of Female and Male Recognition Molecules

Mutation of the gene encoding a female recognition molecule allows the coexistence of a transition allele (Y^F^) and an original allele (Y) over a wide range of values of α. Therefore, the divergence of allorecognition alleles may originate from mutation of the gene encoding the female recognition molecule. In contrast, mutation of the gene encoding the male recognition molecule must be somewhat suppressed, since mutations that inactivate the male recognition molecule reduce the diversity of allorecognition loci, namely, the number of alleles, through selection for the original allele.

In sexual conflict, since the female typically exerts selection on the maleside, the male side undergoes changes to its elements or behaviors to expand the opportunities for mating or to exclude others, whereas the female side changes its elements or behaviors strictly in opposition to the changes on the male side [28,29]. Conversely, as revealed in this study, during the process of allorecognition molecule divergence, mutations in the gene encoding the female recognition molecule occur first, although they increase the risk of self-fertilization, and are not desirable for females. This phenomenon in self-sterility would be an interesting example from the standpoint of sexual conflict, since genes encoding female molecules are mutated first, followed by mutation in the male molecules.

### 4.4. Tolerance to Mutations and Molecular Evolution

In *C. intestinalis*, locus B consists of the tandem genes *s*/*v-Themis-B and -B2* [12]. The ancestral locus is thought to have duplicated. With tandem loci, loss of the allorecognition function through a mutation in one copy can be complemented by the other, although *s*/*v-Themis-B and -B2* are not always linked (manuscript in preparation). The tolerance of mutations in this way may promote the creation of variation in such loci, since mutation is necessary for generating polymorphisms, as shown in this study. In fact, the number of polymorphisms is greater in locus B than in locus A. This finding is consistent with previous statistical research suggesting that mutation and recombination play key roles in the creation and maintenance of polymorphisms in allorecognition loci [30].

## 5. Conclusions

In this research, we introduced a new modeling and simulation approach to investigate the emergence and evolution of allorecognition and observed its consistency with real-world phenomena, such as the coexistence of nonallorecognition allele.

Our study suggests that allorecognition genes, newly generated by mutation, and nonallorecognition allele established a balanced selection state, after which allorecognition diversified to construct the current allorecognition system. We also revealed that mutations in genes encoding male and female allorecognition molecules showed different characteristics and predicted the diversification process of the allorecognition molecules in which female molecules differentiate first.

Although the current study was based on the ascidian *C. intestinalis*, self-incompatibility has also been studied in other animals and plants such as *B. campestris*, and the detailed mechanisms differ among species; for example, both gametophytic and sporophytic mechanisms have been reported in plants. Therefore, we expect that our models could be tailored to different situations in different species to provide novel tools for furthering our knowledge and understanding of allorecognition.

## Figures and Tables

**Table 1:** Comparison of the convergence points from the simulations and analytical solutions (the model for the emergence process).

α	Proportion of the Genotype XX (Simulation After 1000 Generations)	Proportion of the Genotype XX (Analytical Solution)
1.1	1	1
1	0.99802	1
0.9	0.85817	0.85817
0.7	0.71484	0.71484
0.5	0.63122	0.63122
0.3	0.57086	0.57086
0.1	0.52199	0.52199
0.01	0.50215	0.50215

**Table 2:** Comparison of the convergence points from the simulations and analytical solutions for the model with diversified alleles.

M	α	Simulation After 1000 Generations			Analytical Solution		
		*a*	*b*	*c*	*a*	*b*	*c*
5	0.1	0.2193	0.1108	0.0227	0.2193	0.1108	0.0227
5	0.5	0.3128	0.1064	0.0155	0.3128	0.1064	0.0155
5	0.8	0.4734	0.0898	0.0078	0.4738	0.0898	0.0077
10	0.1	0.1468	0.0509	0.0076	0.1465	0.0509	0.0077
10	0.5	0.2165	0.0526	0.0057	0.2158	0.0526	0.0057
10	0.8	0.3529	0.0499	0.0033	0.3496	0.05	0.0033
15	0.5	0.1759	0.034	0.003	0.1722	0.0339	0.003

## Data Availability

The raw data supporting the conclusions of this article will be made available by the authors on request.

## Supplementary Materials

The following supporting information can be downloaded at: https://www.mdpi.com/article/10.3390/biom15101397/s1; Supplementary Material S1: Details of the models for mutation in male or female genes. Supplementary Material S2: Preliminary studies for the model with divergent alleles. Supplementary Material S3: Derivation process of the model with divergent alleles. Supplementary Material S4: Details of the 2-locus model. Supplementary Material S5: Supplemental results for Figure 5. Supplementary Material S6: Verification of the reproducibility of the models. Supplementary Material S7: Phylogenetical analysis for *Themis-B2* alleles.

## Abbreviations

The following abbreviations are used in this manuscript:

allorecognition	allogenic recognition
VC	vitelline coat

## Appendix A. Formulae for Generation Calculations

### Appendix A.1. Models for Mutations in Male or Female Genes

For the model comprising alleles X, Y, and Y^F^, the following equations were derived to calculate the *n*+1-st generation from the *n*-th generation on the basis of the data in Figure 2B,C, and Supplementary Material S1 (Figure S1A).

(A1) $$a_{n+1}^{\prime }=\alpha \left({a_{n}}^{2}+\frac{{c_{n}}^{2}}{4}\right)+\frac{a_{n}\left(b_{n}+2c_{n}\right)}{2}+\frac{\alpha {b_{n}}^{2}+b_{n}\left(2a_{n}+c_{n}\right)}{2\left(2a_{n}+b_{n}+c_{n}\right)}+\frac{b_{n}c_{n}}{4}\;b_{n+1}^{\prime }=\left(\frac{a_{n}}{2}+\frac{c_{n}}{4}\right)\left(b_{n}+d_{n}\right)+\frac{\alpha {b_{n}}^{2}+\left(2a_{n}+c_{n}\right)\left(b_{n}+d_{n}\right)+b_{n}d_{n}}{2\left(2a_{n}+b_{n}+c_{n}\right)}\;c_{n+1}^{\prime }=\frac{\alpha {c_{n}}^{2}}{2}+a_{n}\left(c_{n}+2e_{n}\right)+c_{n}e_{n}+\frac{a_{n}d_{n}+b_{n}e_{n}+d_{n}}{2}+\frac{c_{n}\left(b_{n}+d_{n}\right)}{4}\;d_{n+1}^{\prime }=\left(\frac{c_{n}}{4}+\frac{e_{n}}{2}\right)\left(b_{n}+d_{n}\right)\;e_{n+1}^{\prime }=\alpha \left(\frac{{c_{n}}^{2}}{4}+{e_{n}}^{2}\right)+\frac{e_{n}\left(c_{n}+d_{n}\right)}{2}+\frac{c_{n}\left(d_{n}+{2e}_{n}\right)}{4}$$

In Equation (A1), $a_{n}$–$e_{n}$ indicate the population of each genotype in the *n*-th generation (defined in Figure 2B), and α represents the survival probability.

For the model comprising alleles of X, Y, and Y^M^, the following equations were derived to calculate the *n*+1-st generation from the *n*-th generation on the basis of the data in Figure 2D,E, and Supplementary Material S1 (Figure S1B).

(A2) $$a_{n+1}^{\prime }=\alpha {a_{n}}^{2}+\frac{a_{n}\left(b_{n}+c_{n}\right)}{2}+\frac{\alpha \left({b_{n}}^{2}+{c_{n}}^{2}\right)+2\left(a_{n}b_{n}+a_{n}c_{n}+b_{n}c_{n}\right)}{2\left(2-b_{n}-d_{n}\right)}\;\;b_{n+1}^{\prime }=\frac{a_{n}\left(b_{n}+d_{n}\right)}{2}+\frac{\alpha {b_{n}}^{2}+\left(2a_{n}+c_{n}\right)\left(b_{n}+d_{n}\right)+b_{n}d_{n}}{2\left(2-b_{n}-d_{n}\right)}\;\;c_{n+1}^{\prime }=a_{n}e_{n}+\frac{a_{n}\left(c_{n}+d_{n}\right)}{2}+\frac{2\alpha {c_{n}}^{2}+2\left(a_{n}+b_{n}\right)c_{n}+2\left(a_{n}+{b_{n}+c}_{n}\right)\left(d_{n}+{2e}_{n}\right)}{2\left(2-b_{n}-d_{n}\right)}\;d_{n+1}^{\prime }=\frac{\alpha {d_{n}}^{2}+b_{n}\left(c_{n}+d_{n}+{2e}_{n}\right)+d_{n}\left(c_{n}+{2e}_{n}\right)}{2\left(2-b_{n}-d_{n}\right)}\;\;e_{n+1}^{\prime }=\frac{\alpha \left({c_{n}}^{2}+{d_{n}}^{2}+{4e_{n}}^{2}\right)+4e_{n}\left(c_{n}+d_{n}\right)+2c_{n}d_{n}}{2\left(2-b_{n}-d_{n}\right)}$$

In Equation (A1), $a_{n}$–$e_{n}$ indicate the population of each genotype in the *n*-th generation (defined in Figure 2B), and α represents the survival probability.

For the model comprising alleles of X, Y, Z, and Y^F^, the following equations were derived to calculate the *n*+1-st generation from the *n*-th generation on the basis of the data in Figure 2F,G, and Supplementary Material S1 (Figure S1C).

(A3) $$a_{n+1}^{\prime }=\alpha {a_{n}}^{2}+\frac{a_{n}\left(b_{n}+c_{n}+e_{n}\right)}{2}+\frac{\alpha \left({c_{n}}^{2}+{e_{n}}^{2}\right)+\left(2a_{n}+b_{n}\right)\left(c_{n}+e_{n}\right)+2c_{n}e_{n}}{2\left(2-c_{n}-d_{n}-g_{n}\right)}+\frac{\alpha b^{2}+b_{n}\left(2a_{n}+c_{n}+e_{n}\right)}{2\left(1+a_{n}+c_{n}-f_{n}-h_{n}\right)}\;b_{n+1}^{\prime }=\frac{a_{n}\left(b_{n}+d_{n}+f_{n}\right)+d_{n}+f_{n}}{2}+\frac{\left(c_{n}+e_{n}\right)\left(b_{n}+d_{n}+f_{n}\right)}{2\left(2-c_{n}-d_{n}-g_{n}\right)}+\frac{\alpha b^{2}+b_{n}\left(2a_{n}+c_{n}+e_{n}\right)}{2\left(1+a_{n}+c_{n}-f_{n}-h_{n}\right)}\;\;c_{n+1}^{\prime }=\frac{a_{n}\left(c_{n}+d_{n}+g_{n}\right)+d_{n}}{2}+\frac{\alpha {c_{n}}^{2}+\left(2a_{n}+b_{n}+e_{n}\right)\left(c_{n}+g_{n}\right)+c_{n}g_{n}}{2\left(2-c_{n}-d_{n}-g_{n}\right)}+\frac{b_{n}\left(c_{n}+d_{n}+g_{n}\right)}{2\left(1+a_{n}+c_{n}-f_{n}-h_{n}\right)}\;d_{n+1}^{\prime }=\frac{\left(c_{n}+g_{n}\right)\left(b_{n}+d_{n}+f_{n}\right)}{2\left(2-c_{n}-d_{n}-g_{n}\right)}+\frac{b_{n}\left(c_{n}+d_{n}+g_{n}\right)}{2\left(1+a_{n}+c_{n}-f_{n}-h_{n}\right)}\;\begin{array}{cc} e_{n+1}^{\prime }= & \frac{a_{n}\left(e_{n}+f_{n}+g_{n}+{2h}_{n}\right)+f_{n}}{2}\\ & +\frac{2\alpha {e_{n}}^{2}+\left(c_{n}+e_{n}\right)\left(f_{n}+g_{n}+{2h}_{n}\right)+e_{n}\left({2a}_{n}+b_{n}+2c_{n}\right)+\left(g_{n}+{2h}_{n}\right)\left({2a}_{n}+b_{n}+c_{n}+e_{n}\right)}{2\left(2-c_{n}-d_{n}-g_{n}\right)} \end{array}\;f_{n+1}^{\prime }=\frac{\left(b_{n}+d_{n}+f_{n}\right)\left(e_{n}+g_{n}+{2h}_{n}\right)}{2\left(2-c_{n}-d_{n}-g_{n}\right)}\;g_{n+1}^{\prime }=\frac{\alpha {g_{n}}^{2}+\left(c_{n}+g_{n}\right)\left(e_{n}+f_{n}+{2h}_{n}\right)+c_{n}g_{n}}{2\left(2-c_{n}-d_{n}-g_{n}\right)}\;h_{n+1}^{\prime }=\frac{\alpha \left({e_{n}}^{2}+{g_{n}}^{2}+{{4h}_{n}}^{2}\right)+\left(e_{n}+g_{n}\right)\left(f_{n}+4h_{n}\right)+2e_{n}g_{n}+2f_{n}h_{n}}{2\left(2-c_{n}-d_{n}-g_{n}\right)}$$

In Equation (A3), $a_{n}$–$h_{n}$ indicate the population of each genotype in the *n*-th generation (defined in Figure 2F), and α represents the survival probability.

### Appendix A.2. Model with Divergent Alleles

For the model consisting of a nonallorecognition allele (X) and “M” types of allorecognition alleles (Y_k_s), the characteristics of the X and Y_k_ alleles are described in Figure 3. The following equations were then derived to calculate the *n*+1-st generation from the *n*-th generation on the basis of the data in Figure 3A,B, and Supplementary Material S3.

(A4) $$a_{n+1}^{\prime }=\alpha {a_{n}}^{2}+\frac{Ma_{n}b_{n}}{2}+\frac{M}{2}\cdot \frac{\alpha {b_{n}}^{2}+{2a_{n}b}_{n}+\left(M-1\right){b_{n}}^{2}}{2-b_{n}-\left(M-1\right)c_{n}}\;\;b_{n+1}^{\prime }=\frac{a_{n}b_{n}+\left(M-1\right)a_{n}c_{n}}{2}+\frac{\alpha {b_{n}}^{2}+{2a_{n}b}_{n}+2\left(M-1\right){b_{n}}^{2}+{\left(M-1\right)}^{2}b_{n}c_{n}}{2\left\{2-b_{n}-\left(M-1\right)c_{n}\right\}}+\frac{\left(M-1\right)\left(2{a_{n}c_{n}+Mb}_{n}c_{n}\right)}{2\left\{2-{2b}_{n}-2\left(M-1\right)c_{n}\right\}}\;\;c_{n+1}^{\prime }=\frac{\left(M-1\right)b_{n}c_{n}+{b_{n}}^{2}}{2-b_{n}-\left(M-1\right)c_{n}}+\frac{\left(M-2\right)\left\{b_{n}c_{n}+\left(M-1\right){c_{n}}^{2}\right\}}{2-{2b}_{n}-2\left(M-1\right)c_{n}}$$

In Equation (A4), $a_{n}$–$c_{n}$ indicate the population of each genotype in the *n*-th generation (defined in Figure 3A). α and M represent the survival probability and the number of types of allorecognition alleles respectively.

### Appendix A.3. Model with 2-Loci

For the model with 2 allorecognition loci, the following equations were derived to calculate the *n*+1-st generation from the *n*-th generation on the basis of Supplementary Material S4 (Figure S4A).

(A5) $$\begin{array}{ll} a_{n+1}^{\prime }=\alpha ({a_{n}}^{2}+ & \frac{M{b_{n}}^{2}+L{c_{n}}^{2}}{4}+\frac{LM{d_{n}}^{2}}{4\left(4-P\right)})+a_{n}\left(Mb_{n}+Lc_{n}+\frac{LMd_{n}}{4}\right)+\frac{LMb_{n}c_{n}}{2}\\ & +\frac{M\left(M-1\right){b_{n}}^{2}+L\left(L-1\right){c_{n}}^{2}}{4}+\frac{Mb_{n}+Lc_{n}}{8}LMd_{n}+\frac{2a_{n}+Mb_{n}+Lc_{n}}{2\left(4-P\right)}LMd_{n}\\ & +\frac{LM{{\left(LM-1\right)d}_{n}}^{2}}{4\left(4-P\right)} \end{array}\;\begin{array}{ll} b_{n+1}^{\prime }=\alpha (\frac{{b_{n}}^{2}}{2}+\frac{L{d_{n}}^{2}}{2\left(4-P\right)})+ & a_{n}\left\{b_{n}+\left(M-1\right)g_{n}+2k_{n}\right\}+\left(Mb_{n}+Lc_{n}\right)k_{n}\\ & +\frac{L\left(b_{n}c_{n}+a_{n}l_{n}\right)+\left(M-1\right)\left\{{b_{n}}^{2}+\left(Mb_{n}+Lc_{n}\right)g_{n}\right\}}{2}\\ & +\frac{L\left[a_{n}d_{n}+Lc_{n}l_{n}+M\left\{b_{n}l_{n}+d_{n}\left(b_{n}+k_{n}\right)\right\}\right]}{4}+\frac{L\left(M-1\right)\left\{\left(2a_{n}+Mb_{n}+Lc_{n}\right)h_{n}+Md_{n}g_{n}\right\}}{8}\\ & +\frac{L^{2}c_{n}d_{n}}{8}+\frac{2a_{n}+Mb_{n}+Lc_{n}+LMd_{n}}{4-P}Ll_{n}+\frac{2a_{n}+2M\left(b_{n}+k_{n}\right)+Lc_{n}+M\left(M-1\right)g_{n}}{2\left(4-P\right)}Ld_{n}\\ & +\frac{LM\left(M-1\right)h_{n}+2\left(LM-1\right)d_{n}}{4\left(4-P\right)}Ld_{n}+\frac{L\left(M-1\right)\left(2a_{n}+Mb_{n}+Lc_{n}+0.5LMd_{n}\right)}{4\left(2-P\right)}h_{n} \end{array}\;\begin{array}{ll} c_{n+1}^{\prime }=\alpha (\frac{{c_{n}}^{2}}{2}+ & \frac{M{d_{n}}^{2}}{2\left(4-P\right)})+a_{n}\left\{c_{n}+\left(L-1\right)e_{n}+2i_{n}\right\}+\left(Mb_{n}+Lc_{n}\right)i_{n}\\ & +\frac{M\left(b_{n}c_{n}+a_{n}j_{n}\right)+\left(L-1\right)\left\{{c_{n}}^{2}+\left(Mb_{n}+Lc_{n}\right)e_{n}\right\}}{2}\\ & +\frac{M\left[a_{n}d_{n}+Mb_{n}j+L\left\{c_{n}j_{n}+d_{n}\left(c_{n}+i_{n}\right)\right\}\right]}{4}+\frac{M\left(L-1\right)\left\{\left(2a_{n}+Mb_{n}+Lc_{n}\right)f_{n}+Ld_{n}e_{n}\right\}}{8}\\ & +\frac{M^{2}b_{n}d_{n}}{8}+\frac{2a_{n}+Mb_{n}+Lc_{n}+LMd_{n}}{4-P}Mj_{n}+\frac{2a_{n}+Mb_{n}+2L\left(c_{n}+i_{n}\right)+L\left(L-1\right)e_{n}}{2\left(4-P\right)}Md_{n}\\ & +\frac{LM\left(L-1\right)f_{n}+2\left(LM-1\right)d_{n}}{4\left(4-P\right)}Md_{n}+\frac{M\left(L-1\right)\left(2a_{n}+Mb_{n}+Lc_{n}+0.5LMd_{n}\right)}{4\left(2-P\right)}f_{n} \end{array}\;\begin{array}{ll} d_{n+1}^{\prime }=\frac{3\alpha }{4\left(4-P\right)}{d_{n}}^{2} & +\frac{\left\{2c_{n}+4i_{n}+M\left(d_{n}+{2j}_{n}\right)+\left(L-1\right)\left(2e_{n}+Mf_{n}\right)\right\}\left(b_{n}+\left(M-1\right)g_{n}+2k_{n}\right)}{8}\\ & +\frac{\left\{2b_{n}+4k_{n}+L\left(d_{n}+{2l}_{n}\right)+\left(M-1\right)\left(2g_{n}+Lh_{n}\right)\right\}\left\{c_{n}+\left(L-1\right)e_{n}+2i_{n}\right\}}{8}\\ & +\frac{\left\{d_{n}+2\left(j_{n}+l_{n}\right)+\left(L-1\right)\left(f_{n}+2o_{n}\right)+\left(M-1\right)\left(h_{n}+2n_{n}\right)+\left(L-1\right)\left(M-1\right)m_{n}\right\}}{8}\\ & \cdot \left({2a}_{n}+Mb_{n}+Lc_{n}\right)\\ & +\left\{\frac{d_{n}+{2j}_{n}}{4\left(4-P\right)}+\frac{\left(L-1\right)f_{n}}{8\left(2-P\right)}\right\}\{4a_{n}+4M\left(b_{n}+k_{n}\right)+2Lc_{n}+2LM\left(d_{n}+l_{n}\right)\\ & +M\left(M-1\right)\left(2g_{n}+Lh_{n}\right)\}\\ & +\left\{\frac{d_{n}+{2l}_{n}}{4\left(4-P\right)}+\frac{\left(M-1\right)h_{n}}{8\left(2-P\right)}\right\}\{4a_{n}+2Mb_{n}+4L\left(c_{n}+i_{n}\right)+2LM\left(d_{n}+j_{n}\right)\\ & +L\left(L-1\right)\left(2e_{n}+Mf_{n}\right)\}\\ & +\frac{d_{n}}{4\left(4-P\right)}[-4\left(a_{n}+d_{n}\right)-2\left(Mb_{n}+Lc_{n}\right)\\ & +\left(LM-1\right)\left\{\left(L-1\right)\left(f_{n}+2o_{n}\right)+\left(M-1\right)\left(h_{n}+2n_{n}\right)+2\left(j_{n}+l_{n}\right)+\left(L-1\right)\left(M-1\right)m_{n}\right\}]\\ & +\left\{\frac{\left(L-1\right)\left(M-1\right)m_{n}}{16\left(1-P\right)}+\frac{\left(M-1\right)n_{n}+\left(L-1\right)o_{n}}{4\left(2-P\right)}\right\}\left\{4a_{n}+2\left(Mb_{n}+Lc_{n}\right)+LMd_{n}\right\} \end{array}\;\begin{array}{ll} e_{n+1}^{\prime }=\alpha (\frac{{e_{n}}^{2}}{2} & +\frac{M{f_{n}}^{2}}{2\left(4-P\right)})+2\left(c_{n}+i_{n}\right)i_{n}+Mi_{n}j_{n}+\left(L-1\right)\left(c_{n}+2i_{n}\right)e_{n}+\frac{{c_{n}}^{2}+M\left(c_{n}j_{n}+d_{n}i_{n}\right)}{2}\\ & +\frac{M\left(L-1\right)\left(e_{n}j_{n}+f_{n}i_{n}\right)+L\left(L-2\right){e_{n}}^{2}}{2}+\frac{Mc_{n}d_{n}+M\left(L-1\right)\left\{c_{n}f_{n}+d_{n}e_{n}+\left(L-1\right)e_{n}f_{n}\right\}}{4}\\ & +\frac{M\left[d_{n}\left(c_{n}+2i_{n}\right)+\left(L-1\right)\left(d_{n}e_{n}+Mf_{n}j_{n}\right)+2\left\{c_{n}+Md_{n}+\left(L-1\right)e_{n}+2i_{n}+Mj_{n}\right\}j_{n}\right]}{4-P}\\ & +\frac{M^{2}\left\{d_{n}+\left(L-1\right)f_{n}\right\}}{2\left(4-P\right)}d_{n}+\frac{M\left(L-1\right)\left\{c_{n}+0.5Md_{n}+\left(L-1\right)e_{n}+2i_{n}+Mj_{n}\right\}}{2\left(2-P\right)}f_{n}\\ & +\frac{M\left\{M{\left(L-1\right)}^{2}-1\right\}}{4\left(2-P\right)}{f_{n}}^{2} \end{array}\;\begin{array}{ll} f_{n+1}^{\prime }=\frac{\alpha {f_{n}}^{2}}{4\left(2-P\right)} & +\frac{P\left\{c_{n}+\left(L-1\right)e_{n}+2i_{n}\right\}}{4}+\frac{\left[c_{n}+2i_{n}+M\left\{d_{n}+2\left(j_{n}+l_{n}\right)\right\}\right]\left(d_{n}+2j_{n}\right)}{4-P}\\ & +\frac{\left[\left(L-1\right)\left\{e_{n}+M\left(f_{n}+o_{n}\right)\right\}+M\left(M-1\right)\left\{0.5h_{n}+n_{n}+0.5\left(L-1\right)m_{n}\right\}\right]\left(d_{n}+2j_{n}\right)}{4-P}\\ & +\frac{\left\{2\left(c_{n}+2i_{n}\right)+\left(L-1\right)\left(2e_{n}+Mf_{n}\right)\right\}l_{n}}{4-P}\\ & +\frac{\left\{M\left(L-1\right)-1\right\}\left[d_{n}+\left(M-1\right)\left(h_{n}+2n_{n}\right)+2\left(j_{n}+l_{n}\right)+\left(L-1\right)\left\{\left(M-1\right)m_{n}+2o_{n}\right\}\right]}{4\left(2-P\right)}f_{n}\\ & +\frac{\left\{L\left(L-2\right)+{\left(L-1\right)}^{2}\left(M-1\right)-\left(L-1\right)\right\}}{4\left(2-P\right)}{f_{n}}^{2}\\ & +\left\{\frac{\left(L-1\right)\left(f_{n}+2o_{n}\right)+\left(M-1\right)\left(h_{n}+2n_{n}\right)}{2\left(2-P\right)}+\frac{\left(L-1\right)\left(M-1\right)m_{n}}{4\left(1-P\right)}\right\}\{c_{n}+2i_{n}+M\left(0.5d_{n}+j_{n}\right)\\ & +\left(L-1\right)\left(e_{n}+0.5Mf_{n}\right)\} \end{array}\;\begin{array}{ll} g_{n+1}^{\prime }=\alpha (\frac{{g_{n}}^{2}}{2}+\frac{L{h_{n}}^{2}}{2\left(4-P\right)}) & +2\left(b_{n}+k_{n}\right)k_{n}+Lk_{n}l_{n}+\left(M-1\right)\left(b_{n}+2k_{n}\right)g_{n}+\frac{{b_{n}}^{2}+L\left(b_{n}l_{n}+d_{n}k_{n}\right)}{2}\\ & +\frac{L\left(M-1\right)\left(g_{n}l_{n}+h_{n}k_{n}\right)+M\left(M-2\right){g_{n}}^{2}}{2}\\ & +\frac{Lb_{n}d_{n}+L\left(M-1\right)\left\{b_{n}h_{n}+d_{n}g_{n}+\left(M-1\right)g_{n}h_{n}\right\}}{4}\\ & +\frac{L\left[d_{n}\left(b_{n}+2k_{n}\right)+\left(M-1\right)\left(d_{n}g_{n}+Lh_{n}l_{n}\right)+2\left\{c_{n}+Ld_{n}+\left(M-1\right)g_{n}+2k_{n}+Ll_{n}\right\}l_{n}\right]}{4-P}\\ & +\frac{L^{2}\left\{d_{n}+\left(M-1\right)h_{n}\right\}}{2\left(4-P\right)}d_{n}+\frac{L\left(M-1\right)\left\{b_{n}+0.5Ld_{n}+\left(M-1\right)g_{n}+2k_{n}+Ll_{n}\right\}}{2\left(2-P\right)}h_{n}\\ & +\frac{L\left\{L{\left(M-1\right)}^{2}-1\right\}}{4\left(2-P\right)}{h_{n}}^{2} \end{array}\;\begin{array}{ll} h_{n+1}^{\prime }=\frac{\alpha {h_{n}}^{2}}{4\left(2-P\right)} & +\frac{P\left\{b_{n}+\left(M-1\right)g_{n}+2k_{n}\right\}}{4}+\frac{\left[b_{n}+2k_{n}+L\left\{d_{n}+2\left(j_{n}+l_{n}\right)\right\}\right]\left(d_{n}+2l_{n}\right)}{4-P}\\ & +\frac{\left[\left(M-1\right)\left\{g_{n}+L\left(h_{n}+n_{n}\right)\right\}+L\left(L-1\right)\left\{0.5f_{n}+o_{n}+0.5\left(M-1\right)m_{n}\right\}\right]\left(d_{n}+2l_{n}\right)}{4-P}\\ & +\frac{\left\{2\left(b_{n}+2k_{n}\right)+\left(M-1\right)\left(2g_{n}+Lh_{n}\right)\right\}j_{n}}{4-P}\\ & +\frac{\left\{L\left(M-1\right)-1\right\}\left[d_{n}+\left(L-1\right)\left(f_{n}+2o_{n}\right)+2\left(j_{n}+l_{n}\right)+\left(M-1\right)\left\{\left(L-1\right)m_{n}+2n_{n}\right\}\right]}{4\left(2-P\right)}h_{n}\\ & +\frac{\left\{M\left(M-2\right)+{\left(L-1\right)\left(M-1\right)}^{2}-\left(M-1\right)\right\}}{4\left(2-P\right)}{h_{n}}^{2}\\ & +\left\{\frac{\left(L-1\right)\left(f_{n}+2o_{n}\right)+\left(M-1\right)\left(h_{n}+2n_{n}\right)}{2\left(2-P\right)}+\frac{\left(L-1\right)\left(M-1\right)m_{n}}{4\left(1-P\right)}\right\}\{b_{n}+2k_{n}+L\left(0.5d_{n}+l_{n}\right)\\ & +\left(M-1\right)\left(g_{n}+0.5Lh_{n}\right)\} \end{array}\;\begin{array}{ll} i_{n+1}^{\prime }= & \alpha \{\frac{{c_{n}}^{2}+\left(L-1\right){e_{n}}^{2}}{4}+{i_{n}}^{2}+\frac{M\left({d_{n}}^{2}+{4j_{n}}^{2}\right)}{4\left(4-P\right)}+\frac{M\left(L-1\right){f_{n}}^{2}}{8\left(2-P\right)}\}+c_{n}i_{n}+\left(L-1\right)e_{n}i_{n}+\frac{\left(L-1\right)c_{n}e_{n}}{2}\\ & +\frac{\left(L-1\right)\left(L-2\right)}{4}{e_{n}}^{2}+\frac{M\left\{d_{n}+\left(L-1\right)f_{n}+2j_{n}\right\}\left\{c_{n}+\left(L-1\right)e_{n}+2i_{n}\right\}}{8}\\ & +\frac{M\left[d_{n}i_{n}+\left\{c_{n}+Md_{n}+\left(L-1\right)e_{n}+2i_{n}+\left(M-1\right)j_{n}\right\}j_{n}\right]}{4-P}\\ & +\frac{M\left\{c_{n}d_{n}+\left(L-1\right)\left(d_{n}e_{n}+Mf_{n}j_{n}\right)\right\}}{2\left(4-P\right)}+\frac{M\left\{\left(M-1\right)d_{n}+M\left(L-1\right)f_{n}\right\}d_{n}}{4\left(4-P\right)}\\ & +\frac{M\left(L-1\right)\left[2\left\{c_{n}+M\left(0.5d_{n}+j_{n}\right)+\left(L-1\right)e_{n}+2i_{n}\right\}+\left\{\left(L-1\right)\left(M-1\right)+\left(L-2\right)\right\}f_{n}\right]}{8\left(2-P\right)}f_{n} \end{array}\;\begin{array}{ll} j_{n+1}^{\prime }= & \alpha \left(\frac{{d_{n}}^{2}+4{j_{n}}^{2}}{4\left(4-P\right)}+\frac{\left(L-1\right){f_{n}}^{2}}{8\left(2-P\right)}\right)+\frac{P\left\{c_{n}+\left(L-1\right)e_{n}+2i_{n}\right\}}{8}\\ & +\left\{\frac{l_{n}}{2\left(4-P\right)}+\frac{2\left(L-1\right)o_{n}+\left(M-1\right)\left(h_{n}+2n_{n}\right)}{8\left(2-P\right)}+\frac{\left(L-1\right)\left(M-1\right)m_{n}}{16\left(1-P\right)}\right\}\{{2c}_{n}+4i_{n}\\ & +M\left(d_{n}+2j_{n}\right)+\left(L-1\right)\left(2e_{n}+Mf_{n}\right)\}\\ & +\left\{\frac{d_{n}+2j_{n}}{4\left(4-P\right)}+\frac{\left(L-1\right)f_{n}}{8\left(2-P\right)}\right\}[{2c}_{n}+4i_{n}+\left(M-1\right)\left(d_{n}+l_{n}\right)+\left(L-1\right)\left(2e_{n}+Mf_{n}\right)\\ & +{\left(M-1\right)}^{2}\left(h_{n}+2n_{n}\right)+\left(L-1\right)\left(M-1\right)\left\{f_{n}+2o_{n}+\left(M-1\right)m_{n}\right\}+2\left(2M-1\right)j_{n}]\\ & +\frac{\left(d_{n}-2j_{n}\right)j_{n}}{2\left(4-P\right)}+\frac{\left(L-1\right){\left(d_{n}-f_{n}\right)f}_{n}}{8\left(2-P\right)} \end{array}\;\begin{array}{ll} k_{n+1}^{\prime }= & \alpha \left\{\frac{{b_{n}}^{2}+\left(M-1\right){g_{n}}^{2}}{4}+{k_{n}}^{2}+\frac{L\left({d_{n}}^{2}+{4l_{n}}^{2}\right)}{4\left(4-P\right)}+\frac{L\left(M-1\right){h_{n}}^{2}}{8\left(2-P\right)}\right\}+b_{n}k_{n}+\left(M-1\right)g_{n}k_{n}+\frac{\left(M-1\right)b_{n}g_{n}}{2}\\ & +\frac{\left(M-1\right)\left(M-2\right)}{4}{g_{n}}^{2}+\frac{L\left\{d_{n}+\left(M-1\right)h_{n}+2l_{n}\right\}\left\{b_{n}+\left(M-1\right)g_{n}+2k_{n}\right\}}{8}\\ & +\frac{L\left[d_{n}k_{n}+\left\{b_{n}+Ld_{n}+\left(M-1\right)g_{n}+2k_{n}+\left(L-1\right)l_{n}\right\}l_{n}\right]}{4-P}\\ & +\frac{L\left\{b_{n}d_{n}+\left(M-1\right)\left(d_{n}g_{n}+Lh_{n}l_{n}\right)\right\}}{2\left(4-P\right)}+\frac{L\left\{\left(L-1\right)d_{n}+L\left(M-1\right)h_{n}\right\}d_{n}}{4\left(4-P\right)}\\ & +\frac{L\left(M-1\right)\left[2\left\{b_{n}+L\left(0.5d_{n}+l_{n}\right)+\left(M-1\right)g_{n}+2k_{n}\right\}+\left\{\left(L-1\right)\left(M-1\right)+\left(M-2\right)\right\}h_{n}\right]}{8\left(2-P\right)}h_{n} \end{array}\;\begin{array}{ll} l_{n+1}^{\prime }= & \alpha \left(\frac{{d_{n}}^{2}+4{l_{n}}^{2}}{4\left(4-P\right)}+\frac{\left(M-1\right){h_{n}}^{2}}{8\left(2-P\right)}\right)+\frac{P\left\{b_{n}+\left(M-1\right)g_{n}+2k_{n}\right\}}{8}\\ & +\left\{\frac{j_{n}}{2\left(4-P\right)}+\frac{2\left(M-1\right)n_{n}+\left(L-1\right)\left(f_{n}+2o_{n}\right)}{8\left(2-P\right)}+\frac{\left(L-1\right)\left(M-1\right)m_{n}}{16\left(1-P\right)}\right\}\{{2b}_{n}+4k_{n}\\ & +L\left(d_{n}+2l_{n}\right)+\left(M-1\right)\left(2g_{n}+Lh_{n}\right)\}\\ & +\left\{\frac{d_{n}+2l_{n}}{4\left(4-P\right)}+\frac{\left(M-1\right)h_{n}}{8\left(2-P\right)}\right\}[{2b}_{n}+4k_{n}+\left(L-1\right)\left(d_{n}+j_{n}\right)+\left(M-1\right)\left(2g_{n}+Lh_{n}\right)\\ & +{\left(L-1\right)}^{2}\left(f_{n}+2o_{n}\right)+\left(L-1\right)\left(M-1\right)\left\{h_{n}+2n_{n}+\left(L-1\right)m_{n}\right\}+2\left(2L-1\right)l_{n}]\\ & +\frac{\left(d_{n}-2l_{n}\right)l_{n}}{2\left(4-P\right)}+\frac{\left(M-1\right){\left(d_{n}-h_{n}\right)h}_{n}}{8\left(2-P\right)} \end{array}\;m_{n+1}^{\prime }=P\left\{\frac{d_{n}+2\left(j_{n}+l_{n}\right)}{4-P}+\frac{\left(L-1\right)\left(f_{n}+2o_{n}\right)+\left(M-1\right)\left(h_{n}+2n_{n}\right)}{2\left(2-P\right)}+\frac{\left(LM-L-M\right)m_{n}}{4\left(1-P\right)}\right\}\;n_{n+1}^{\prime }=P\left\{\frac{d_{n}+2\left(j_{n}+l_{n}\right)}{2\left(4-P\right)}+\frac{\left(L-1\right)\left(f_{n}+2o_{n}\right)+\left(M-2\right)\left(h_{n}+2n_{n}\right)}{4\left(2-P\right)}+\frac{\left(L-1\right)\left(M-2\right)m_{n}}{8\left(1-P\right)}\right\}\;o_{n+1}^{\prime }=P\left\{\frac{d_{n}+2\left(j_{n}+l_{n}\right)}{2\left(4-P\right)}+\frac{\left(L-2\right)\left(f_{n}+2o_{n}\right)+\left(M-1\right)\left(h_{n}+2n_{n}\right)}{4\left(2-P\right)}+\frac{\left(L-2\right)\left(M-1\right)m_{n}}{8\left(1-P\right)}\right\}\;\mathrm{Here}\;P\;\mathrm{is}\;\mathrm{defined}\;\mathrm{as};\;\;P=d_{n}+\left(L-1\right)\left(f_{n}+2o_{n}\right)+\left(M-1\right)\left(h_{n}+2n_{n}\right)+2\left(j_{n}+l_{n}\right)+\left(L-1\right)\left(M-1\right)m_{n}$$

In Equation (A5), $a_{n}$–$o_{n}$ indicate the population of each genotype in the *n*-th generation (defined in Figure 4B), and α represents the survival probability. L and M represent the numbers of types of allorecognition alleles in loci A and B respectively.
