# Supplementary material for: Population Genetics of the Emergence and Evolution of Allogenic Recognition During Fertilization
Source: Biomolecules. 2025 Sep 30;15(10):1397. doi: 10.3390/biom15101397 (PMC12562240; doi:10.3390/biom15101397)
Supplement: Supplementary file 1 [file biomolecules-15-01397-s001.zip › biomolecules-3876266-supplementary.pdf]

Supplementary Material S1 Derivation process of the models for mutation in male or female genes

To model the situation consisting of X, Y, and Y^F alleles (Figs. 2B and C), the numbers of next-generation individuals produced by each combination of gametes can be expressed as shown in Fig. S1A. From this, Equation (A1) is derived.

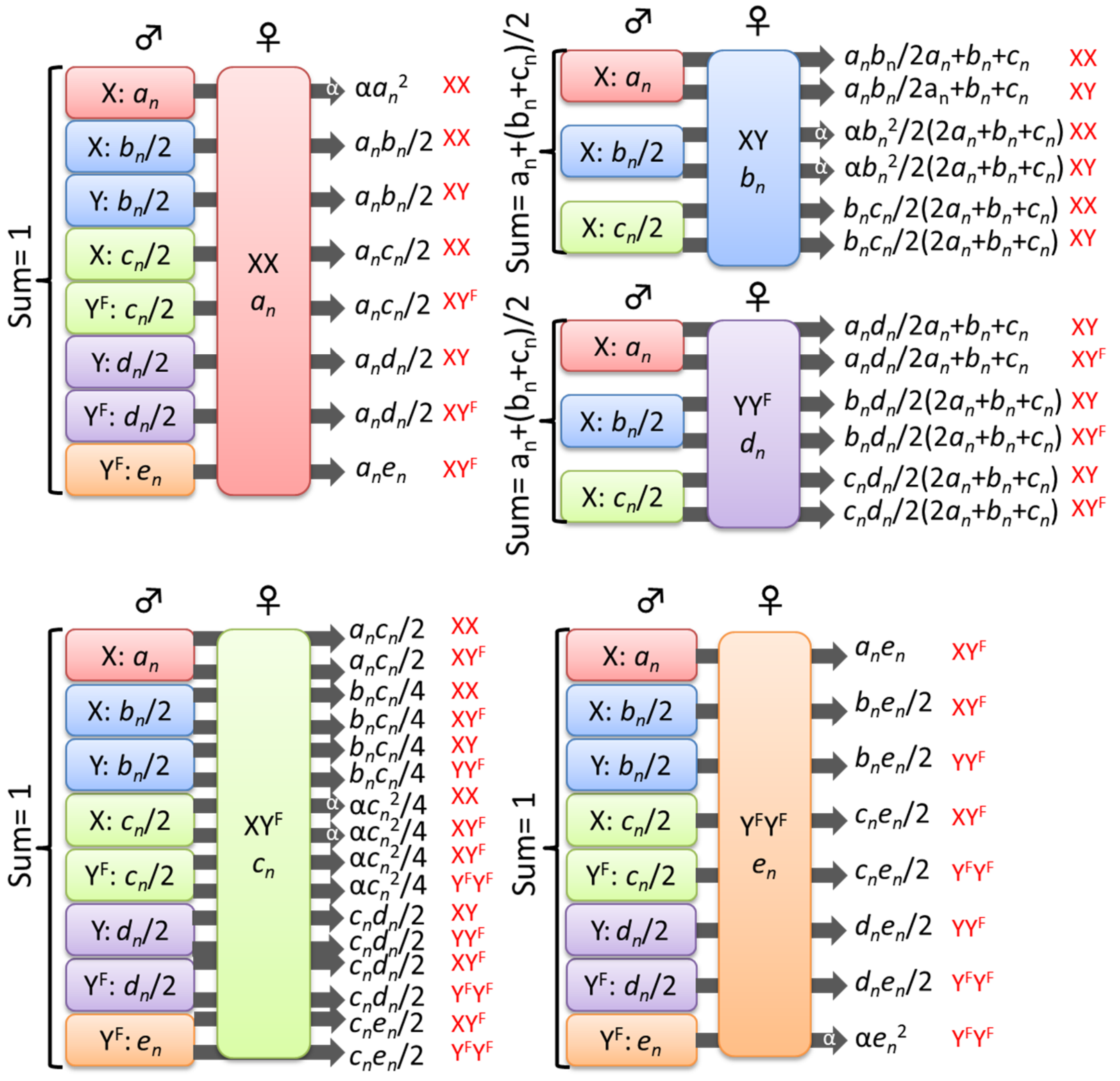

Supplementary Figure S1A. Calculations of the next generation in the model, comprising alleles X, Y, and Y^F.

To model the situation consisting of X, Y, and Y^M alleles (Figs. 2D and E), the numbers of next-generation individuals produced by each combination of gametes can be expressed as shown in Fig. S1B. From this, Equation (A2) is derived.

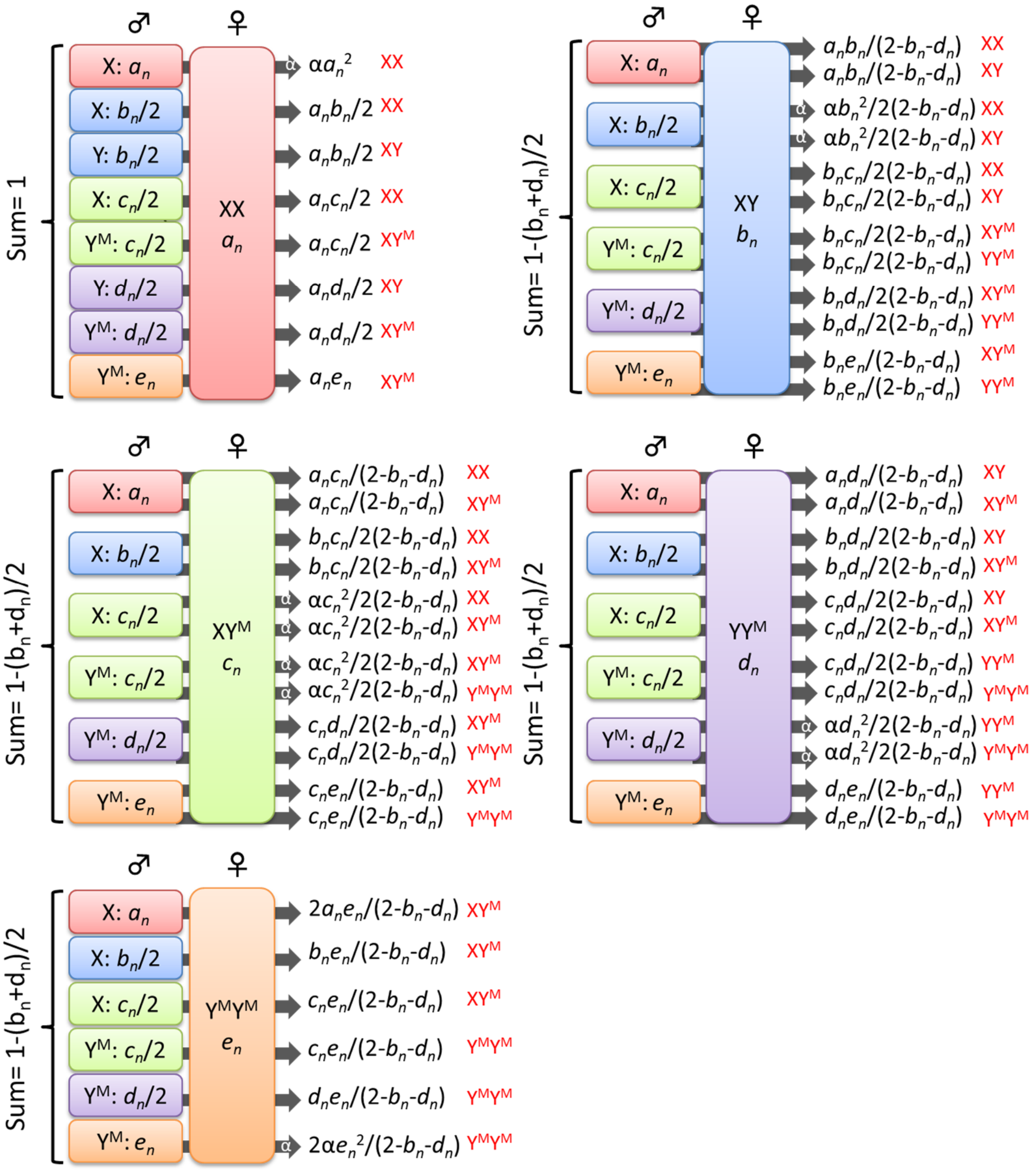

Supplementary Figure S1B. Calculations of the next generation in the model, comprising alleles X, Y, and Y^M.

To model the situation consisting of X, Y, Z, and Y^F alleles (Figs. 2F and G), the numbers of next-generation individuals produced by each combination of gametes can be expressed as shown in Fig. S1C. From this, Equation (A3) is derived.

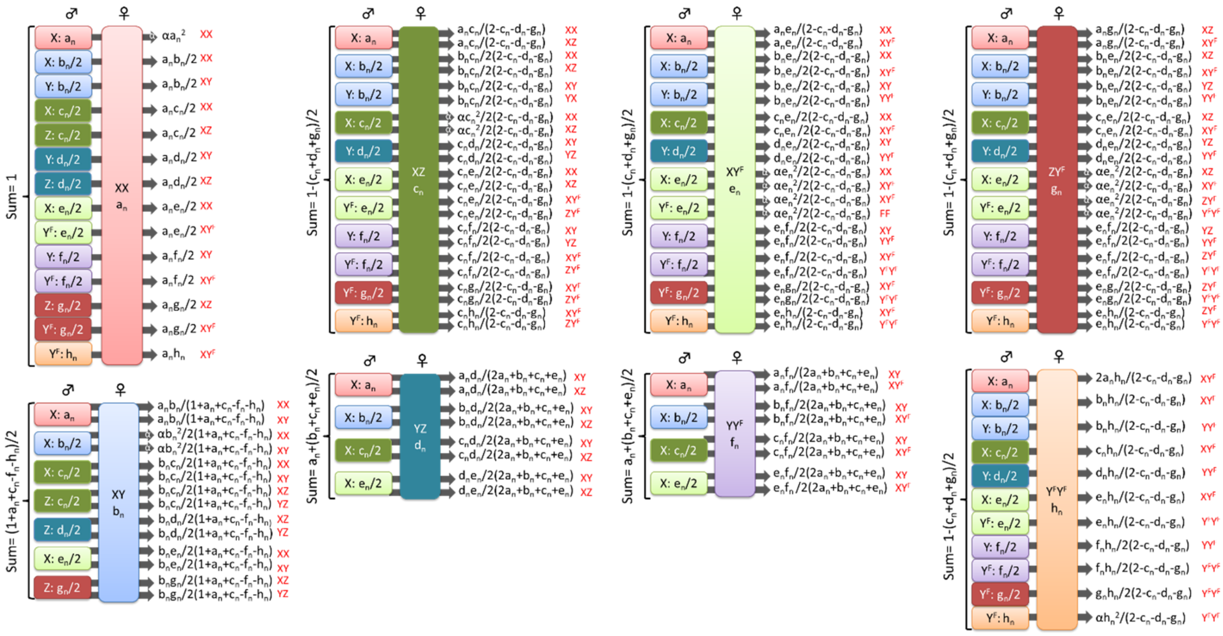

Supplementary Figure S1C. Calculations of the next generation in the model, comprising alleles X, Y, Z, and Y^F.

Supplementary Material S2 Preliminary studies for the model with divergent alleles

M=2 Model

To model the situation in which the number of allorecognition alleles increases, a preliminary study in which two allorecognition alleles coexist with a nonallorecognition allele ($M = 2$) was conducted. Fig. S2A shows the mating characteristics of the $M = 2$ genotypes. The number of next-generation individuals produced by each combination of gametes can be expressed as shown in Fig. S2B. From this, Equation (S2a) was derived to calculate the *n+1*-st generation from the *n*-th generation. Figs. S2C and S2D show the representative results of simulations with the $M = 2$ model.

Genotype / Proportion	XX / a_n	XY_1 / b^1_n	XY_2 / b^2_n	Y_1Y_2 / c_n
Ratio of sperm (♂) genotype	X: a_n	X: $b^1_n/2$<br>Y_1: $b^1_n/2$	X: $b^2_n/2$<br>Y_2: $b^2_n/2$	Y_1: $c_n/2$<br>Y_2: $c_n/2$
Proportion of egg (♀) genotype	XX: a_n	XY_1: b^1_n	XY_2: b^2_n	Y_1Y_2: c_n

Egg genotype

Sperm genotype	XX	XY_1	XY_2	Y_1Y_2
X	✓	✓	✓	✓
Y_1	✓	✖	✓	✖
Y_2	✓	✓	✖	✖

Supplementary Figure S2A. Mating characteristics of the $M = 2$ model.

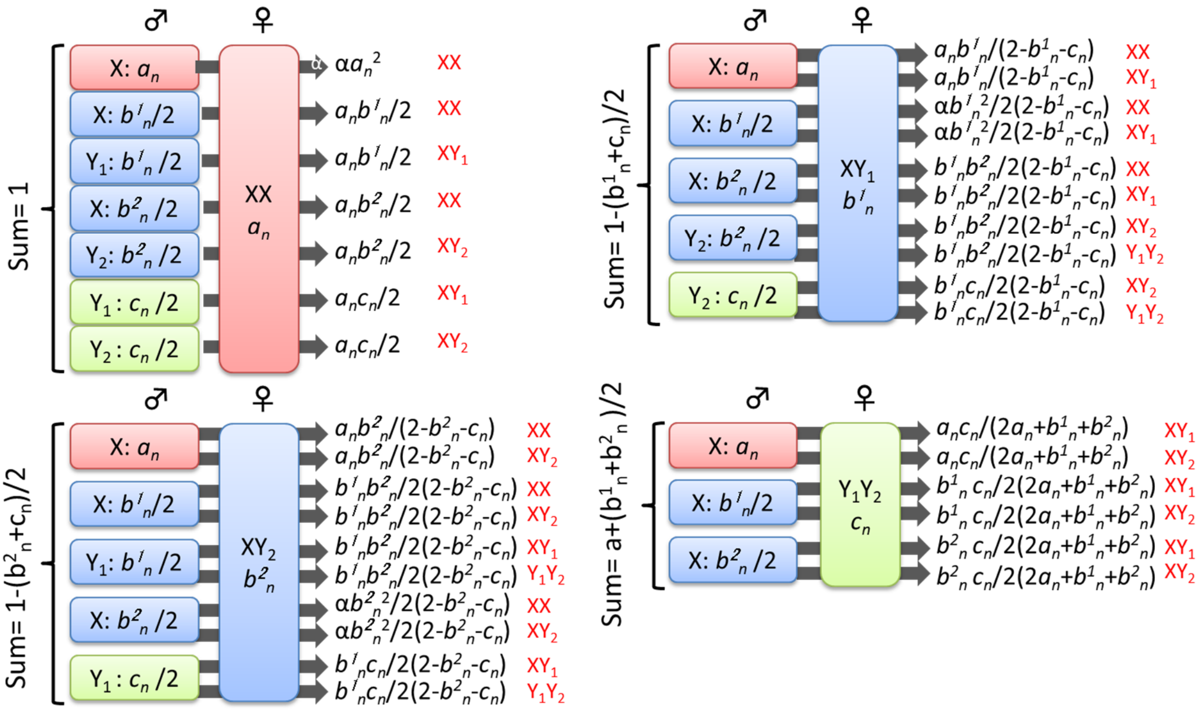

Supplementary Figure S2B. Calculations of the next generation in the $M = 2$ model.

$$
\begin{aligned}
a'_{n+1} &= \alpha a_n{}^2 + \frac{a_n(b^1{}_n + b^2{}_n)}{2} + \frac{\alpha b^1{}_n{}^2 + b^1{}_n(2a_n + b^2{}_n)}{2(2 - b^1{}_n - c_n)} + \frac{\alpha b^2{}_n{}^2 + b^2{}_n(2a_n + b^1{}_n)}{2(2 - b^2{}_n - c_n)} \\
b^{1\prime}{}_{n+1} &= \frac{a_n(b^1{}_n + c_n)}{2} + \frac{\alpha b^1{}_n{}^2 + b^1{}_n(2a_n + b^2{}_n)}{2(2 - b^1{}_n - c_n)} + \frac{b^2{}_n(b^1{}_n + c_n)}{2(2 - b^2{}_n - c_n)} \\
b^{2\prime}{}_{n+1} &= \frac{a_n(b^2{}_n + c_n)}{2} + \frac{b^1{}_n(b^2{}_n + c_n)}{2(2 - b^1{}_n - c_n)} + \frac{\alpha b^2{}_n{}^2 + b^2{}_n(2a_n + b^1{}_n)}{2(2 - b^2{}_n - c_n)} \\
c'_{n+1} &= \frac{b^1{}_n(b^2{}_n + c_n)}{2(2 - b^1{}_n - c_n)} + \frac{b^2{}_n(b^1{}_n + c_n)}{2(2 - b^2{}_n - c_n)}
\end{aligned}
\qquad \text{(S2a)}
$$

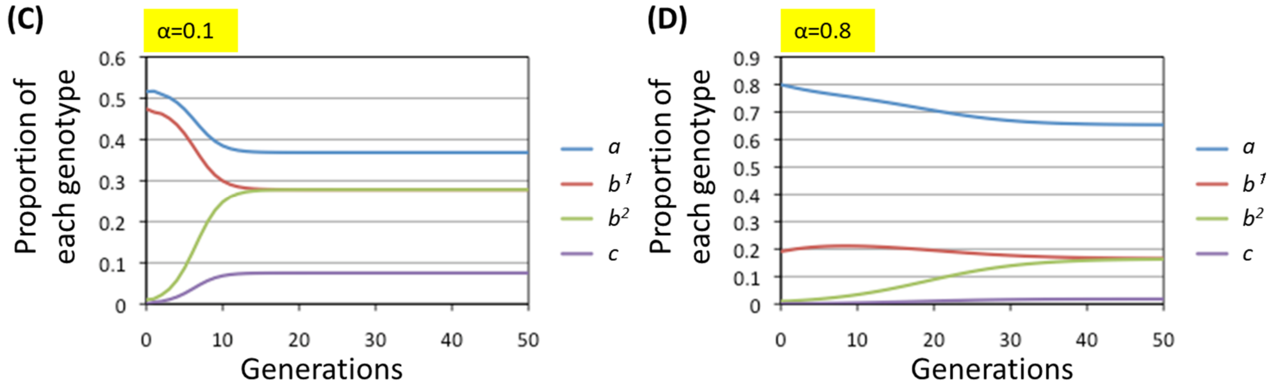

Supplementary Figure S2C-D. Representative results of the $M = 2$ model. Proportions of each genotype (vertical axis) were plotted against the number of generations (horizontal axis). Heteropopulations of X with Y_1 or Y_2 (b^1 and b^2) converged to the same proportion after multiple generations, although the initial proportions were different.

M=3 Model

To model the situation in which the number of allorecognition alleles increases, a preliminary study in the case of $M = 3$ was conducted. Fig. S2E shows the mating characteristics of the $M = 3$ genotypes. The number of next-generation individuals produced by each combination of gametes can be expressed as shown in Fig. S2F. From this, Equation (S2b) was derived to calculate the *n+1*-st generation from the *n*-th generation. Figs. S2G and S2H show the representative results of simulations with the $M = 3$ model.

Genotype / Proportion	XX / a_n	XY_1 / b^1_n	XY_2 / b^2_n	XY_3 / b^3_n	Y_1Y_2 / c^1_n	Y_1Y_3 / c^2_n	Y_2Y_3 / c^3_n
Ratio of sperm (♂) genotype	X: a_n	X: $b^1_n/2$<br>Y_1: $b^1_n/2$	X: $b^2_n/2$<br>Y_2: $b^2_n/2$	X: $b^3_n/2$<br>Y_2: $b^3_n/2$	Y_1: $c^1_n/2$<br>Y_2: $c^1_n/2$	Y_1: $c^2_n/2$<br>Y_3: $c^2_n/2$	Y_2: $c^3_n/2$<br>Y_3: $c^3_n/2$
Proportion of egg (♀) genotype	XX: a_n	XY_1: b^1_n	XY_2: b^2_n	XY_3: b^3_n	Y_1Y_2: c^1_n	Y_1Y_3: c^2_n	Y_2Y_3: c^3_n

Egg genotype

Sperm genotype	XX	XY_1	XY_2	XY_3	Y_1Y_2	Y_1Y_3	Y_2Y_3
X	✓	✓	✓	✓	✓	✓	✓
Y_1	✓	✖	✓	✓	✖	✖	✓
Y_2	✓	✓	✖	✓	✖	✓	✖
Y_3	✓	✓	✓	✖	✓	✖	✖

Supplementary Figure S2E. Mating characteristics of the $M = 3$ model.

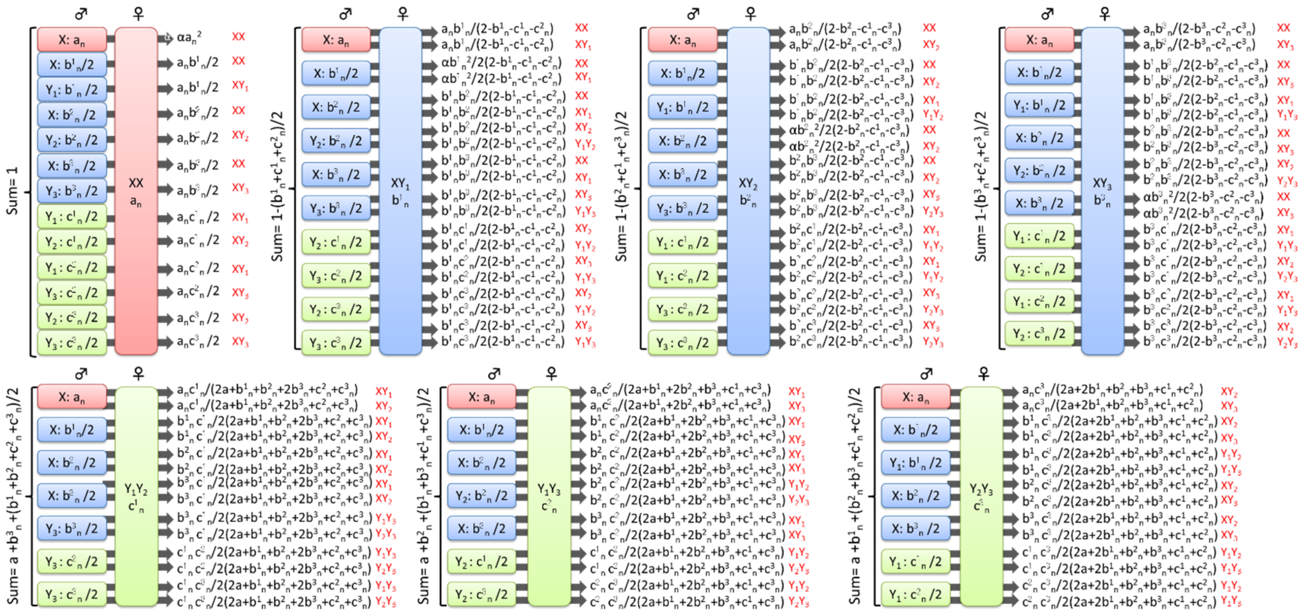

Supplementary Figure S2F. Calculations of the next generation in the $M = 3$ model.

$$\begin{aligned}
a'_{n+1} = {} & \alpha\left\{{a_n}^2 + 0.5\left(\frac{{{b^1}_n}^2}{2-{b^1}_n-{c^1}_n-{c^2}_n} + \frac{{{b^2}_n}^2}{2-{b^2}_n-{c^1}_n-{c^3}_n} + \frac{{{b^3}_n}^2}{2-{b^3}_n-{c^2}_n-{c^3}_n}\right)\right\} \\
& + 0.5a_n({b^1}_n+{b^2}_n+{b^3}_n) + \frac{(a_n+{b^2}_n+{b^3}_n){b^1}_n}{2(2-{b^1}_n-{c^1}_n-{c^2}_n)} + \frac{(a_n+{b^1}_n+{b^3}_n){b^2}_n}{2(2-{b^2}_n-{c^1}_n-{c^3}_n)} \\
& + \frac{(a_n+{b^2}_n+{b^3}_n){b^3}_n}{2(2-{b^3}_n-{c^2}_n-{c^3}_n)} \\
{b^1}'_{n+1} = {} & \frac{\alpha{{b^1}_n}^2}{2(2-{b^1}_n-{c^1}_n-{c^2}_n)} + 0.5\left(a_n + \frac{{b^2}_n}{2-{b^2}_n-{c^1}_n-{c^3}_n} + \frac{{b^3}_n}{2-{b^3}_n-{c^2}_n-{c^3}_n}\right)({b^1}_n+{c^1}_n+{c^2}_n) \\
& + \frac{(2a_n+{b^2}_n+{b^3}_n){b^1}_n}{2(2-{b^1}_n-{c^1}_n-{c^2}_n)} + \frac{(2a_n+{b^1}_n+{b^2}_n+{b^3}_n){c^1}_n}{2(2a_n+{b^1}_n+{b^2}_n+2{b^3}_n+{c^2}_n+{c^3}_n)} \\
& + \frac{(2a_n+{b^1}_n+{b^2}_n+{b^3}_n){c^2}_n}{2(2a_n+{b^1}_n+2{b^2}_n+{b^3}_n+{c^1}_n+{c^3}_n)} \\
{b^2}'_{n+1} = {} & \frac{\alpha{{b^2}_n}^2}{2(2-{b^2}_n-{c^1}_n-{c^3}_n)} + 0.5\left(a_n + \frac{{b^1}_n}{2-{b^1}_n-{c^1}_n-{c^2}_n} + \frac{{b^3}_n}{2-{b^3}_n-{c^2}_n-{c^3}_n}\right)({b^2}_n+{c^1}_n+{c^3}_n) \\
& + \frac{(2a_n+{b^1}_n+{b^3}_n){b^2}_n}{2(2-{b^2}_n-{c^1}_n-{c^3}_n)} + \frac{(2a_n+{b^1}_n+{b^2}_n+{b^3}_n){c^1}_n}{2(2a_n+{b^1}_n+{b^2}_n+2{b^3}_n+{c^2}_n+{c^3}_n)} \\
& + \frac{(2a_n+{b^1}_n+{b^2}_n+{b^3}_n){c^3}_n}{2(2a_n+2{b^1}_n+{b^2}_n+{b^3}_n+{c^1}_n+{c^2}_n)} \\
{b^3}'_{n+1} = {} & \frac{\alpha{{b^3}_n}^2}{2(2-{b^3}_n-{c^2}_n-{c^3}_n)} + 0.5\left(a_n + \frac{{b^1}_n}{2-{b^1}_n-{c^1}_n-{c^2}_n} + \frac{{b^2}_n}{2-{b^2}_n-{c^1}_n-{c^3}_n}\right)({b^3}_n+{c^2}_n+{c^3}_n) \\
& + \frac{(2a_n+{b^1}_n+{b^2}_n){b^3}_n}{2(2-{b^3}_n-{c^2}_n-{c^3}_n)} + \frac{(2a_n+{b^1}_n+{b^2}_n+{b^3}_n){c^2}_n}{2(2a_n+{b^1}_n+2{b^2}_n+{b^3}_n+{c^1}_n+{c^3}_n)} \\
& + \frac{(2a_n+{b^1}_n+{b^2}_n+{b^3}_n){c^3}_n}{2(2a_n+2{b^1}_n+{b^2}_n+{b^3}_n+{c^1}_n+{c^2}_n)} \\
{c^1}'_{n+1} = {} & \frac{{b^2}_n+{c^1}_n+{c^3}_n}{2}\left(\frac{{b^1}_n}{2-{b^1}_n-{c^1}_n-{c^2}_n} + \frac{{c^2}_n}{2a_n+{b^1}_n+2{b^2}_n+{b^3}_n+{c^1}_n+{c^3}_n}\right) \\
& + \frac{{b^1}_n+{c^1}_n+{c^2}_n}{2}\left(\frac{{b^2}_n}{2-{b^2}_n-{c^1}_n-{c^3}_n} + \frac{{c^3}_n}{2a_n+2{b^1}_n+{b^2}_n+{b^3}_n+{c^1}_n+{c^2}_n}\right) \\
{c^2}'_{n+1} = {} & \frac{{b^3}_n+{c^2}_n+{c^3}_n}{2}\left(\frac{{b^1}_n}{2-{b^1}_n-{c^1}_n-{c^2}_n} + \frac{{c^1}_n}{2a_n+{b^1}_n+{b^2}_n+2{b^3}_n+{c^2}_n+{c^3}_n}\right) \\
& + \frac{{b^1}_n+{c^1}_n+{c^2}_n}{2}\left(\frac{{b^3}_n}{2-{b^3}_n-{c^2}_n-{c^3}_n} + \frac{{c^3}_n}{2a_n+2{b^1}_n+{b^2}_n+{b^3}_n+{c^1}_n+{c^2}_n}\right) \\
{c^3}'_{n+1} = {} & \frac{{b^3}_n+{c^2}_n+{c^3}_n}{2}\left(\frac{{b^2}_n}{2-{b^2}_n-{c^1}_n-{c^3}_n} + \frac{{c^1}_n}{2a_n+{b^1}_n+{b^2}_n+2{b^3}_n+{c^2}_n+{c^3}_n}\right) \\
& + \frac{{b^2}_n+{c^1}_n+{c^3}_n}{2}\left(\frac{{b^3}_n}{2-{b^3}_n-{c^2}_n-{c^3}_n} + \frac{{c^2}_n}{2a_n+{b^1}_n+2{b^2}_n+{b^3}_n+{c^1}_n+{c^3}_n}\right)
\end{aligned} \tag{S2b}$$

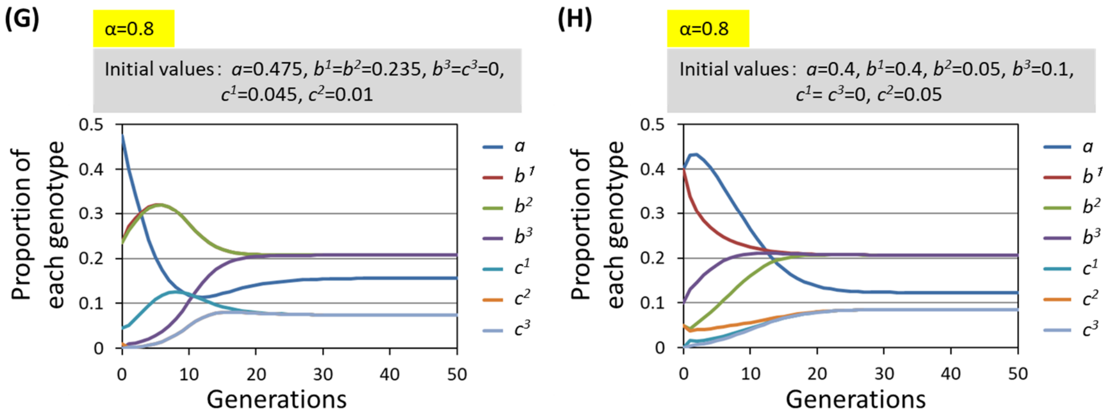

Supplementary Figure S2G-H Representative results of the $\mathrm{M} = 3$ model. Proportions of each genotype (vertical axis) were plotted against the number of generations (horizontal axis). Heterogenotypic populations consisting of two different Y alleles (c^1, c^2 and c^3) converged to the same proportion after multiple generations, as did heterogenotypic populations of X with Y_1, Y_2 or Y_3 (b^1, b^2 and b^3).

Supplementary Material S3 Derivation process of the model with divergent alleles

For the model consisting of a nonallorecognition allele (X) and M types of allorecognition alleles (Y_ks), the number of next-generation individuals produced by each combination of gametes can be expressed as shown in Fig. S3. From this, Equation (A4) is derived.

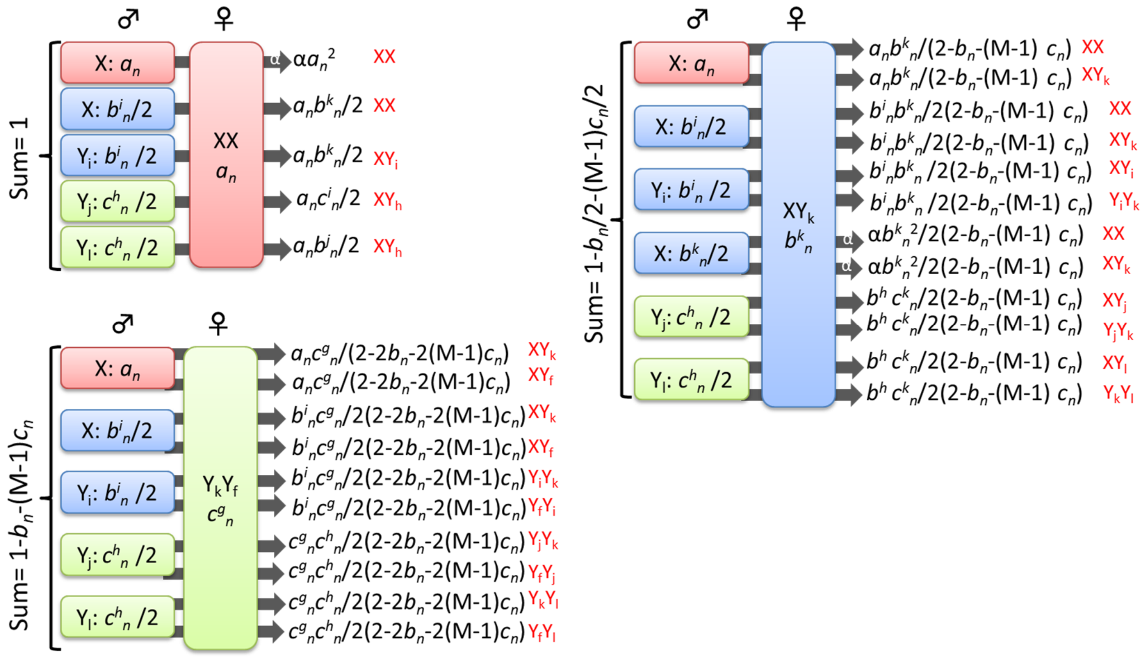

Supplementary Figure S3. Calculations of the next generation in the model with diversified alleles.

Supplementary Material S4 Details of the 2-Locus Model

To model the situation with two allorecognition loci, genotypes and alleles were defined as shown in Fig. 4B, and their characteristics are shown in Fig. S4A. From this, Equation (A5) is derived through the calculation of the next generation.

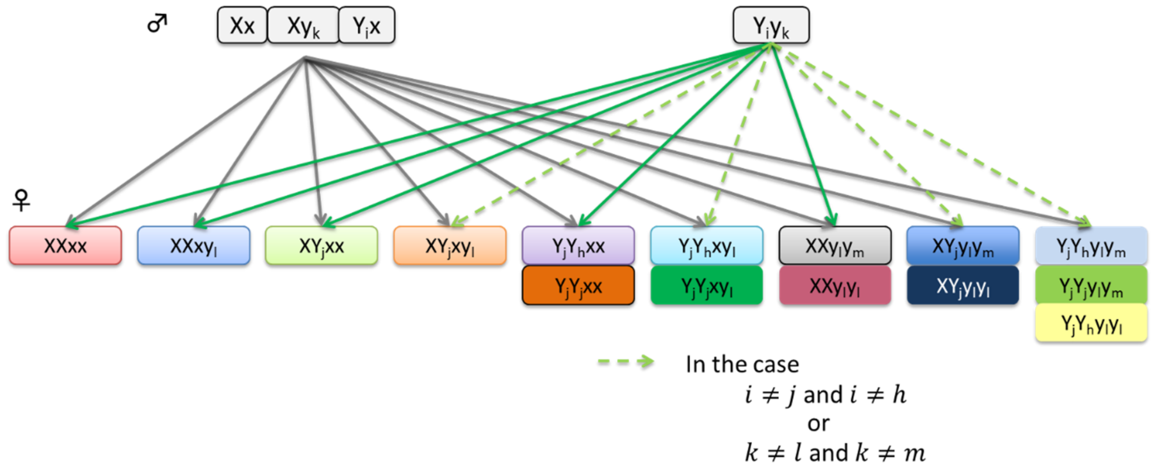

Supplementary Figure S4A. Mating characteristics of the 2-locus model.

To simulate the situation in which the nonallorecognition allele has been eliminated at one locus (Locus A) and remains only at the other locus (Locus B), genotypes and alleles were defined as shown in Fig. S4B, and their characteristics are shown in Fig. S4C. Afterward, the calculations between generations were conducted using Equation (A5), where the initial proportions of individuals a, b, c, d, g, h, k, and l were set to zero.

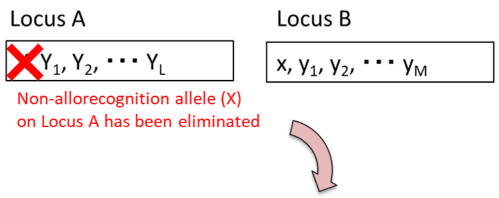

Genotype (Locus A / Locus B)										
		Y$_i$Y$_j$ (hetero) xx	Y$_i$Y$_j$ (hetero) xy$_M$		Y$_i$Y$_i$ (homo) xx	Y$_i$Y$_i$ (homo) xy$_k$		Y$_i$Y$_j$ (hetero) y$_k$y$_l$ (hetero)	Y$_i$Y$_i$ (homo) y$_k$y$_l$ (hetero)	Y$_i$Y$_j$ (hetero) y$_k$y$_k$ (homo)
Proportion		e_n	f_n		i_n	j_n		m_n	n_n	o_n
The number of types		$_LC_2$	$_LC_2 \times M$		L	$L \times M$		$_LC_2 \times {_MC_2}$	$_MC_2 \times L$	$_LC_2 \times M$

Supplementary Figure S4B. Description of the model in which the nonallorecognition allele has been eliminated at one locus (Locus A) and remains at the other locus (Locus B).

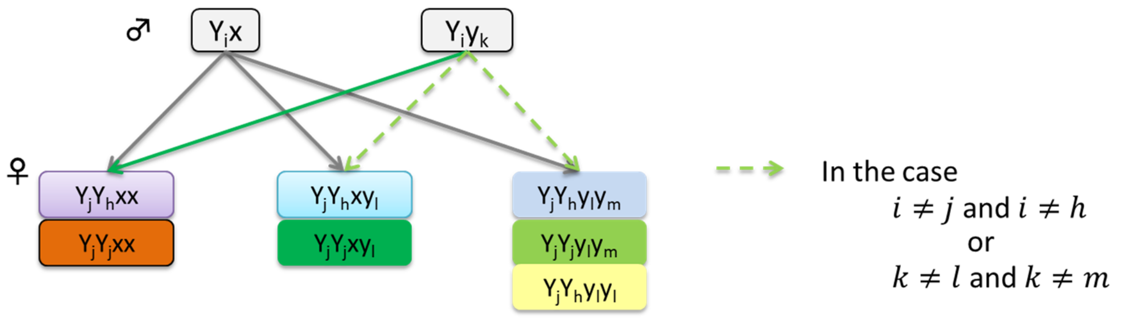

Supplementary Figure S4C. Mating characteristics of the model.

The summarized results are shown in Figs. S4D-G. In all the cases with varied numbers of L and M alleles, the nonallorecognition allele (x) coexisted with the allorecognition alleles (y) in $\alpha < 1$ even after multiple generations.

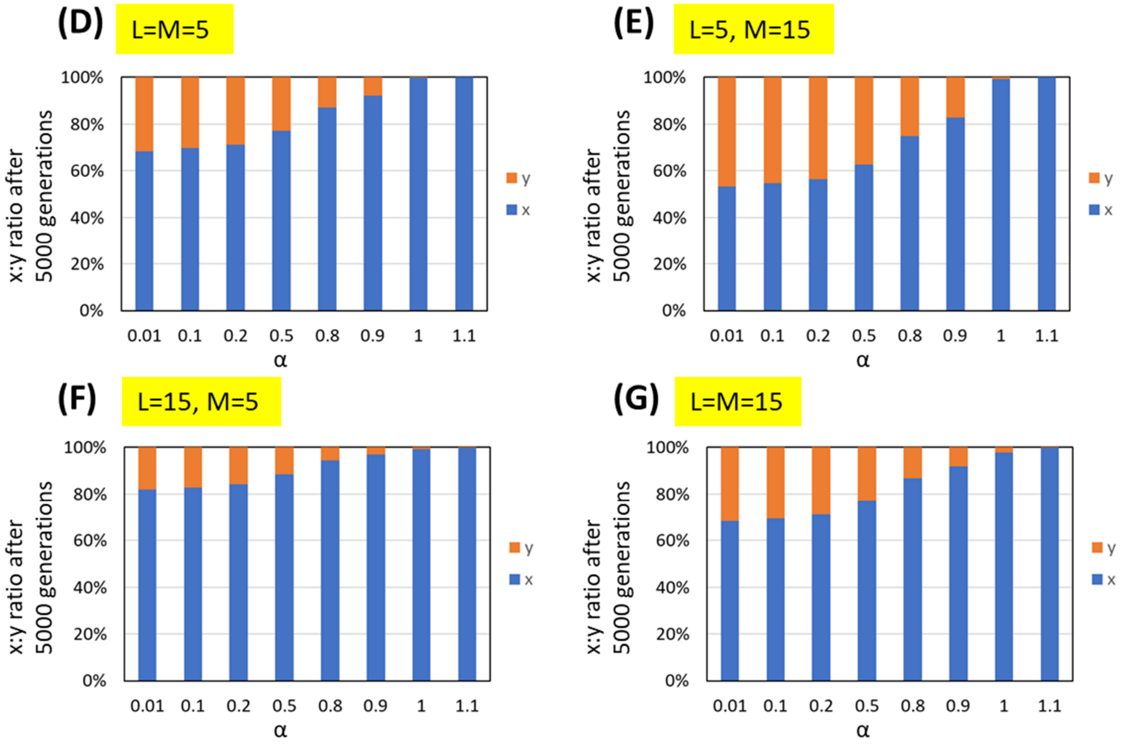

Supplementary Figure S4D-G. Summarized results of the simulations. The ratio of x vs. y (vertical axis) was plotted against the value of α (horizontal axis). Here, y represents the total proportion of allorecognition alleles. In all conditions, the nonallorecognition allele (x) did not show any tendency to disappear.

Supplementary Material S5 Supplemental results for Fig. 5

The supplemental results for Fig. 5 show that the proportions of XY individuals converged to a value dependent on only α regardless of b_0 after multiple generations. Fig. S5 shows representative results of the simulation.

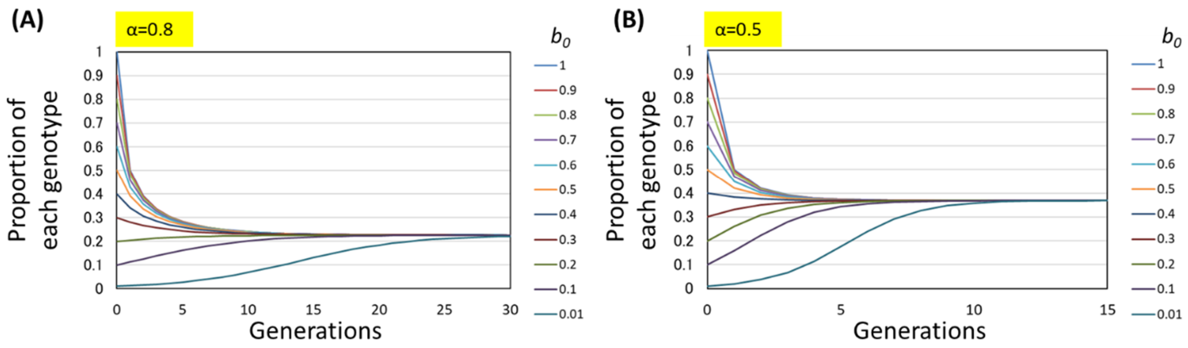

Supplementary Figure S5. Representative results of the simulation for the emersion process of the allorecognition locus. The proportions of XY individuals (vertical axis) were plotted against the number of generations (horizontal axis). b_0 was varied between 0. 01 and 1 in both cases. (**A**) Results of the cases where α was fixed at 0.8. In all cases, the proportions of XY individuals converged to the same value of approximately 0. 22 after multiple generations. (**B**) Results of the cases where α was fixed at 0. 5. In all cases, the proportions of XY individuals converged to the same value of approximately 0.37 after multiple generations.

As shown in Table S1, the simulation using randomized initial conditions resulted in almost no deviation, indicating the reproducibility of the model.

Supplementary Table S1. Verification of the reproducibility of the model. Ten simulation runs were conducted for each α value.

**α**	**Proportion of the genotype XX after 100 generations (N=10)**	
	Average	Standard deviation
0.9	0.85817	2.71202×10^{-6}
0.7	0.71484	9.93014×10^{-17}
0.5	0.63122	1.11022×10^{-16}
0.3	0.57086	1.11022×10^{-16}
0.1	0.52199	8.59975×10^{-17}
0.01	0.50215	1.11022×10^{-16}

Supplementary Material S6 Verification of the reproducibility of the models

To verify the reproducibility and robustness of the convergence points, simulations with randomized initial conditions were conducted using each model.

Table S2A shows a summary of simulations using the model, comprising alleles X, Y, and Y^F. Simulations with randomized initial conditions resulted in extremely small deviations at the convergence points, indicating the reproducibility of the model.

Supplementary Table S2A. Verification of the reproducibility of the model, comprising alleles X, Y, and Y^F.

α	Proportions of each genotype after 1,000 generations (N=10)				
	a	*b*	*c*	*d*	*e*
0.1	0.47 $\pm 1.1 \times 10^{-16}$	0.37 $\pm 5.6 \times 10^{-17}$	0.14 $\pm 2.0 \times 10^{-17}$	0.020 $\pm 2.5 \times 10^{-18}$	0.0022 $\pm 4.3 \times 10^{-19}$
0.5	0.47 $\pm 1.1 \times 10^{-16}$	0.37 $\pm 5.6 \times 10^{-17}$	0.14 $\pm 2.0 \times 10^{-17}$	0.020 $\pm 2.5 \times 10^{-18}$	0.0022 $\pm 4.3 \times 10^{-19}$
0.9	0.83 $\pm 2.8 \times 10^{-4}$	0.13 $\pm 1.0 \times 10^{-4}$	0.038 $\pm 3.7 \times 10^{-4}$	0.0014 $\pm 1.3 \times 10^{-5}$	0.00038 $\pm 7.5 \times 10^{-6}$

Table S2B shows a summary of simulations using the model, comprising alleles X, Y, and Y^M. The simulations resulted in the convergence to almost the same value (0 in "*b*" and "*d*") and almost no deviation in "*a*", "*c*", and "*e*", indicating the reproducibility of the model.

Supplementary Table S2B. Verification of the reproducibility of the model, comprising alleles X, Y, and Y^M.

α	Proportions of each genotype after 1,000 generations (N=10)				
	a	*b*	*c*	*d*	*e*
0.1	0.22 $\pm 9.7 \times 10^{-11}$	9.0×10^{-13} $\pm 2.6 \times 10^{-13}$	0.57 $\pm 2.9 \times 10^{-13}$	6.2×10^{-13} $\pm 1.8 \times 10^{-13}$	0.22 $\pm 9.7 \times 10^{-11}$
0.5	0.23 $\pm 2.3 \times 10^{-16}$	6.6×10^{-114} $\pm 1.0 \times 10^{-113}$	0.53 $\pm 8.6 \times 10^{-17}$	4.5×10^{-114} $\pm 7.1 \times 10^{-114}$	0.23 $\pm 2.4 \times 10^{-16}$
0.9	0.25 $\pm 9.2 \times 10^{-7}$	5.15×10^{-168} ± 0	0.51 $\pm 8.1 \times 10^{-12}$	3.5×10^{-168} ± 0	0.24 $\pm 9.2 \times 10^{-7}$

Table S6C shows a summary of simulations using the model, comprising alleles X, Y, Z, and Y^F. Simulations with randomized initial conditions resulted in quite small deviations at the convergence points, indicating the reproducibility of the model.

Supplementary Table 2C. Verification of the reproducibility of the model, comprising alleles X, Y, Z, and Y^F.

α	Proportions of each genotype after 1,000 generations (N=10)			
	a	b	c	d
0.1	0.37<br>$\pm 4.6 \times 10^{-17}$	0.22<br>$\pm 7.5 \times 10^{-17}$	0.26<br>± 0	0.056<br>$\pm 2.2 \times 10^{-17}$
0.5	0.49<br>$\pm 2.0 \times 10^{-16}$	0.18<br>$\pm 4.0 \times 10^{-15}$	0.22<br>$\pm 6.6 \times 10^{-16}$	0.034<br>$\pm 8.3 \times 10^{-16}$
0.9	0.76<br>$\pm 1.3 \times 10^{-5}$	0.087<br>$\pm 1.4 \times 10^{-3}$	0.11<br>$\pm 6.6 \times 10^{-5}$	0.0059<br>$\pm 1.0 \times 10^{-4}$

α	Proportions of each genotype after 1,000 generations (N=10)			
	e	f	g	h
0.1	0.070<br>$\pm 6.8 \times 10^{-17}$	0.0074<br>$\pm 6.3 \times 10^{-18}$	0.0093<br>$\pm 8.5 \times 10^{-18}$	0.00092<br>$\pm 1.9 \times 10^{-18}$
0.5	0.061<br>$\pm 4.5 \times 10^{-15}$	0.0046<br>$\pm 2.4 \times 10^{-16}$	0.0058<br>$\pm 4.1 \times 10^{-16}$	0.0010<br>$\pm 1.5 \times 10^{-16}$
0.9	0.029<br>$\pm 1.6 \times 10^{-3}$	0.00077<br>$\pm 2.9 \times 10^{-5}$	0.0010<br>$\pm 5.3 \times 10^{-5}$	0.00025<br>$\pm 2.5 \times 10^{-5}$

Table S6D shows a summary of simulations using the M=3 model. Simulations with randomized initial conditions resulted in almost no deviations at the convergence points, indicating the reproducibility of the model. Although the initial populations were random, b^1, b^2, and b^3 converged to the same population reproducibly; c^1, c^2, and c^3 as well.

Supplementary Table 2D. Verification of the reproducibility of the M=3 model.

α	Proportions of each genotype after 500 generations (N=10)						
	a	b^1	b^2	b^3	c^1	c^2	c^3
0.1	0.092<br>$\pm 3.6 \times 10^{-17}$	0.20<br>$\pm 1.8 \times 10^{-17}$	0.20<br>$\pm 1.8 \times 10^{-17}$	0.20<br>$\pm 1.8 \times 10^{-17}$	0.099<br>$\pm 4.4 \times 10^{-17}$	0.099<br>$\pm 4.4 \times 10^{-17}$	0.099<br>$\pm 4.4 \times 10^{-17}$
0.5	0.12<br>$\pm 2.8 \times 10^{-17}$	0.21<br>$\pm 2.6 \times 10^{-17}$	0.21<br>$\pm 2.6 \times 10^{-17}$	0.21<br>$\pm 2.6 \times 10^{-17}$	0.085<br>$\pm 9.8 \times 10^{-18}$	0.085<br>$\pm 9.8 \times 10^{-18}$	0.085<br>$\pm 9.8 \times 10^{-18}$
0.9	0.17<br>$\pm 8.3 \times 10^{-17}$	0.21<br>$\pm 2.0 \times 10^{-17}$	0.21<br>$\pm 2.0 \times 10^{-17}$	0.21<br>$\pm 2.0 \times 10^{-17}$	0.070<br>$\pm 2.6 \times 10^{-17}$	0.070<br>$\pm 2.6 \times 10^{-17}$	0.070<br>$\pm 2.6 \times 10^{-17}$

Table S2E shows a summary of simulations using the model with diversified alleles. Simulations with randomized initial conditions resulted in almost no deviations at the convergence points, indicating the reproducibility of the model.

Supplementary Table 2E. Verification of the reproducibility of the model with diversified alleles.

M	α	Simulation after 1,000 generations (N=10)		
		a	*b*	*c*
5	0.1	0.22 $\pm 2.8 \times 10^{-17}$	0.11 ± 0	0.023 $\pm 5.1 \times 10^{-18}$
5	0.5	0.31 $\pm 6.1 \times 10^{-17}$	0.11 ± 0	0.015 $\pm 3.2 \times 10^{-18}$
5	0.9	0.60 $\pm 2.7 \times 10^{-16}$	0.072 $\pm 3.8 \times 10^{-17}$	0.0040 $\pm 5.7 \times 10^{-18}$
10	0.1	0.15 $\pm 4.4 \times 10^{-17}$	0.051 $\pm 5.8 \times 10^{-18}$	0.0077 $\pm 1.6 \times 10^{-18}$
10	0.5	0.22 $\pm 1.2 \times 10^{-16}$	0.053 $\pm 6.6 \times 10^{-18}$	0.0057 $\pm 2.7 \times 10^{-18}$
10	0.9	0.47 $\pm 2.8 \times 10^{-16}$	0.044 $\pm 2.2 \times 10^{-17}$	0.0020 $\pm 2.8 \times 10^{-18}$
15	0.1	0.12 $\pm 1.1 \times 10^{-16}$	0.032 $\pm 6.6 \times 10^{-18}$	0.0039 $\pm 2.3 \times 10^{-18}$
15	0.5	0.17 $\pm 7.9 \times 10^{-17}$	0.034 $\pm 6.6 \times 10^{-18}$	0.0030 $\pm 1.3 \times 10^{-18}$
15	0.9	0.40 $\pm 4.3 \times 10^{-16}$	0.032 $\pm 1.3 \times 10^{-17}$	0.0012 $\pm 2.3 \times 10^{-18}$

Table S2F shows a summary of simulations using the 2-locus model. Simulations with randomized initial values a_0, b_0, c_0, and d_0 resulted in almost no deviations at the convergence points, indicating the reproducibility of the model.

Supplementary Table 2F. Verification of the reproducibility of the 2-locus model.

L	M	b_0, c_0	α	Proportions of each genotype after 10,000 generations (N=10)						
				a	*b*	*c*	*d*	*e*	*f*	*g*
9	9	$b_0 > c_0$	0.5	0.011 $\pm 7.3 \times 10^{-9}$	0.015 $\pm 3.5 \times 10^{-9}$	0.00078 $\pm 7.1 \times 10^{-10}$	0.0011 $\pm 4.8 \times 10^{-10}$	2.8×10^{-5} $\pm 3.2 \times 10^{-11}$	3.9×10^{-5} $\pm 2.5 \times 10^{-11}$	0.011 $\pm 2.4 \times 10^{-9}$
		$b_0 = c_0$		0.044 ± 0	0.012 $\pm 1.7 \times 10^{-18}$	0.012 $\pm 1.7 \times 10^{-18}$	0.0031 ± 0	0.0016 $\pm 2.2 \times 10^{-19}$	0.0004 $\pm 5.4 \times 10^{-20}$	0.0016 $\pm 2.2 \times 10^{-19}$
		$b_0 < c_0$		0.011 $\pm 1.1 \times 10^{-7}$	0.00078 $\pm 1.0 \times 10^{-8}$	0.015 $\pm 5.0 \times 10^{-8}$	0.0011 $\pm 7.0 \times 10^{-9}$	0.011 $\pm 3.5 \times 10^{-8}$	0.00075 $\pm 2.0 \times 10^{-10}$	2.8×10^{-5} $\pm 4.6 \times 10^{-10}$
3	7	$b_0 = c_0$	0.1	0.015 $\pm 8.3 \times 10^{-17}$	0.024 $\pm 3.8 \times 10^{-17}$	0.0030 $\pm 2.2 \times 10^{-17}$	0.0044 $\pm 1.6 \times 10^{-17}$	0.00027 $\pm 2.6 \times 10^{-18}$	0.00039 $\pm 2.3 \times 10^{-18}$	0.018 $\pm 4.1 \times 10^{-17}$
			0.5	0.016 $\pm 3.5 \times 10^{-18}$	0.027 ± 0	0.0018 ± 0	0.0030 ± 0	0.0001 $\pm 1.4 \times 10^{-20}$	0.00017 ± 0	0.022 $\pm 3.5 \times 10^{-18}$
			0.9	0.016 $\pm 8.6 \times 10^{-12}$	0.030 $\pm 6.6 \times 10^{-12}$	0.00042 $\pm 3.6 \times 10^{-13}$	0.0008 $\pm 4.1 \times 10^{-13}$	5.7×10^{-6} $\pm 6.5 \times 10^{-15}$	1.1×10^{-5} $\pm 8.7 \times 10^{-15}$	0.029 $\pm 3.1 \times 10^{-12}$

**L**	**M**	b_0, c_0	**α**	**Proportions of each genotype after 10,000 generations (N=10)**							
				h	*i*	*j*	*k*	*l*	*m*	*n*	*o*
9	9	$b_0 > c_0$	0.5	0.0075 $\pm 1.4 \times 10^{-11}$	1.4×10^{-5} $\pm 1.5 \times 10^{-11}$	1.8×10^{-5} $\pm 1.2 \times 10^{-11}$	0.0051 $\pm 1.2 \times 10^{-9}$	0.00037 $\pm 7.7 \times 10^{-12}$	2.7×10^{-5} $\pm 5.1 \times 10^{-12}$	1.2×10^{-5} $\pm 2.4 \times 10^{-12}$	1.3×10^{-5} $\pm 2.4 \times 10^{-12}$
		$b_0 = c_0$		0.0004 $\pm 5.4 \times 10^{-20}$	0.00075 $\pm 1.1 \times 10^{-19}$	0.00019 ± 0	0.00075 $\pm 1.1 \times 10^{-19}$	0.00019 ± 0	5.1×10^{-5} $\pm 6.8 \times 10^{-21}$	2.4×10^{-5} ± 0	2.4×10^{-5} ± 0
		$b_0 < c_0$		3.9×10^{-5} $\pm 3.7 \times 10^{-10}$	0.0051 $\pm 1.7 \times 10^{-8}$	0.00037 $\pm 1.1 \times 10^{-10}$	1.4×10^{-5} $\pm 2.2 \times 10^{-10}$	1.8×10^{-5} $\pm 1.8 \times 10^{-10}$	2.7×10^{-5} $\pm 7.4 \times 10^{-11}$	1.3×10^{-5} $\pm 3.5 \times 10^{-11}$	1.2×10^{-5} $\pm 3.5 \times 10^{-11}$
3	7	$b_0 = c_0$	0.1	0.0032 $\pm 3.4 \times 10^{-19}$	0.00012 $\pm 1.2 \times 10^{-18}$	0.00018 $\pm 1.1 \times 10^{-18}$	0.0085 $\pm 1.8 \times 10^{-17}$	0.0015 $\pm 1.7 \times 10^{-19}$	0.00028 $\pm 6.4 \times 10^{-19}$	0.00012 $\pm 3.1 \times 10^{-19}$	0.00013 $\pm 2.8 \times 10^{-19}$
			0.5	0.0024 $\pm 4.3 \times 10^{-19}$	4.9×10^{-5} $\pm 1.4 \times 10^{-20}$	7.8×10^{-5} $\pm 1.4 \times 10^{-20}$	0.010 $\pm 1.7 \times 10^{-18}$	0.0012 $\pm 2.2 \times 10^{-19}$	0.00013 $\pm 2.7 \times 10^{-20}$	5.9×10^{-5} ± 0	6.4×10^{-5} ± 0
			0.9	0.00076 $\pm 1.4 \times 10^{-13}$	2.8×10^{-6} $\pm 3.2 \times 10^{-15}$	5.0×10^{-6} $\pm 3.2 \times 10^{-15}$	0.014 $\pm 1.5 \times 10^{-12}$	0.00038 $\pm 7.0 \times 10^{-14}$	1.0×10^{-5} $\pm 4.9 \times 10^{-15}$	4.4×10^{-6} $\pm 2.2 \times 10^{-15}$	5.0×10^{-6} $\pm 2.4 \times 10^{-15}$

Supplementary Material S7 Phylogenetical analysis for *Themis-B2* alleles

To analyze whether the B2-3 allele is simply a transient allele due to a recent mutation or sustainably coexists with others in the context of balancing selection, a phylogenetic analysis of B2 alleles was carried out. Fig. S7 shows the phylogenetic tree drawn from six B2 alleles using *Themis-B2* gene sequences. In the phylogenetic tree, the B2-3 allele is not particularly close to any of the other alleles, indicating that the B2-3 allele has been independent for many generations.

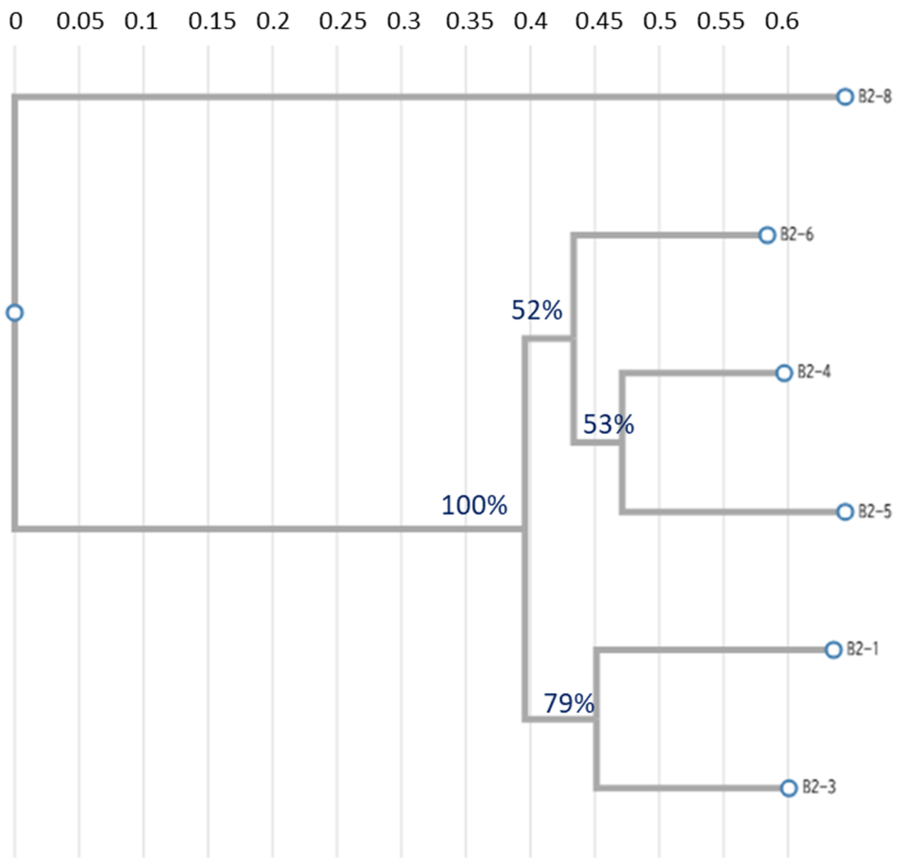

Supplementary Figure S7. Phylogenetic tree of *Themis-B2* gene sequences. The horizontal axis indicates the branch length, and the numbers on the nodes represent the bootstrap probability. A phylogenetic tree was constructed by the maximum likelihood method in ClustalW (https://www.genome.jp/tool-bin/clustalw). The following sequences (accession numbers) were used for this analysis: B2-1 (LC425354), B2-3 (LC425356), B2-4 (LC425357), B2-5 (LC425358), B2-6 (LC425359), and B2-8 (LC425351).
